# Heuristic multi-site optimization for protein sequence design using Masked Protein Language Models

**DOI:** 10.1371/journal.pcbi.1014365

**Published:** 2026-06-05

**Authors:** Lijuan Wang, Yuze Wang, Chen Qiu, Liwei Xiao, Xianliang Liu, Junjie Chen

**Affiliations:** 1 School of Computer Science and Technology, Harbin Institute of Technology, Shenzhen, Guangdong, China; 2 School of Computer Science and Technology, Harbin Institute of Technology, Weihai, Shandong, China; Zhengzhou University of Light Industry, CHINA

## Abstract

Protein sequence design for tailored functional properties is a fundamental task in protein engineering, with critical applications in drug discovery and therapeutic development. Efficient navigation of the combinatorial vastness of protein sequence space to identify functional variants remains a formidable challenge. Conventional approaches, which predominantly rely on template-based local search or single-residue mutagenesis, are constrained by their susceptibility to local optima and their potential risk of destabilizing native structural stability. In this study, we introduce ProtHMSO, a heuristic multi-site optimization framework leveraging masked protein language models (ProtLMs) for context-aware sequence exploration. ProtHMSO mimics natural evolutionary mechanisms by employing ProtLM-derived substitution probabilities to guide heuristic searches for synergistic mutations, thereby constraining combinatorial search spaces through evolutionary and biophysical priors. ProtHMSO is further applied to replace the exploration strategies in genetic algorithms (GAs) and Monte Carlo tree search (MCTS) for improving their convergence efficiency. Benchmark experiments demonstrate that protein sequences generated by ProtHMSO exhibit superior functional performance and closer alignment with natural sequence distribution, compared with state-of-the-art methods. These advancements highlight that ProtHMSO has strong potential and compatibility to accelerate functional protein discovery, offering a robust framework for efficient and context-aware exploration of protein sequence space.

## Author summary

To address the challenge of efficiently discovering functional new proteins in protein engineering due to the vast sequence space, and to overcome the limitations of traditional evolutionary algorithms that rely on blind random mutagenesis, resulting in inefficiency and prone to structural destabilization, we proposed a heuristic multi-site optimization framework, ProtHMSO. Its core concept is to leverage the powerful contextual prediction capabilities of masked protein language models (such as ESM-2) to guide sequence mutagenesis. By predicting amino acid substitutions at specific sites that are consistent with evolutionary laws and biophysical priors, ProtHMSO narrows the exploration scope from the vast combinatorial space to a small number of high-potential candidate sequences, achieving intelligent and efficient optimization of protein sequences. Furthermore, ProtHMSO is not just a standalone algorithm, but also a plug-and-play enhancement module. By integrating it into a genetic algorithm (GA) and a Monte Carlo tree search (MCTS), it replaces the random mutation operator in the former with its intelligent mutation and guides the tree expansion process in the latter. This enables these classic optimization algorithms to break free from the blindness of exploration and achieve faster convergence and better results, demonstrating the wide applicability and great potential of this framework in improving the performance of tools in the entire field of computational protein design.

## Introduction

Protein design aims to discover high-fitness sequences with tailored functional attributes, such as enzymatic activity, thermodynamic stability, and binding affinity [1,2], by exploring astronomically vast fitness landscapes. The directed evolution (DE) framework is a cornerstone of protein sequence optimization through iterative cycles of random mutagenesis, high-throughput screening, and selective pressure to optimize sequences [3,4]. While DE has enabled breakthroughs in enzyme engineering [5–7] and antibody design [8–10], it is constrained by its dependence on experimental screening. This limitation is further exacerbated by the combinatorial explosion of sequence space, which makes exhaustive search infeasible. For instance, a 100-residue protein corresponds to a design space of 20^100^ (∼10^130^) possible variants. As fitness requirements increase, functional sequences become exponentially rarer [11,12], necessitating computational strategies to mitigate combinatorial explosion.

To address these challenges, various computational strategies based on evolutionary algorithms have been proposed. Genetic algorithms (GAs) [13] simulate evolutionary processes through mutation, crossover, and selection. Early applications, such as GA-driven synthesis of bioactive compounds [14,15] and directed evolution of hexapeptides [16], demonstrated efficiency of evolutionary algorithms via iterative optimization. However, GAs struggle with diversity and efficiency in long-sequence optimization [14,17] due to random mutagenesis in the large combinatorial space, which contains vast invalid sequences. Hybrid machine learning (ML)-evolutionary approaches partially address this by guiding mutations to reduce blind exploration. For instance, Yoshida et al. [18] combined a generalized linear model with GA to predict the activity effects of amino acid substitutions, enabling guided mutations in antimicrobial peptide optimization. Zhang et al. [19] narrowed the mutation space by integrating multiple algorithms (FCM clustering, PLSR regression, Monte Carlo simulation), and subsequently screened high-frequency mutations to obtain highly active antimicrobial peptides. Nevertheless, the improvement of optimized sequences was highly dependent on the quality of natural peptides, and scalability to long sequences or complex sequences remains limited. In order to further strengthen the exploration of innovative sequences and reduce dependence on natural sequences, EvoPlay framework [20] employed Monte Carlo Tree Search (MCTS), taking single-point mutation action and policy value neural network to guide protein sequence optimization. PMMG [21] introduce Pareto-optimized multi-property design, enabling the generation of diverse protein sequences. These GAs and MCTS-based approaches lack context-aware mutation guidance, leading to blindness and inefficiency of mutation.

Deep learning approaches leverage structural information or functional attributes to seek high-potential mutations [22–24]. This strategy makes the exploration focus on few important sites, therefore largely reducing the exploration space. For instance, AB-Gen [25] integrated generative pre-trained Transformer (GPT) and deep reinforcement learning (RL) for antibody CDRH3 optimization under multi-attribute constraints. DeepDirect [26] employed adversarial learning to discover affinity mutation sites. MMSite [27] leveraged multimodal fusion for revealing active site identification. ProMEP [28] demonstrated zero-shot mutation effect prediction via sequence-structure representation learning. Despite these advances, efficiently navigating the multi-site mutation space remains a bottleneck. A major hurdle is the fitness landscape’s ruggedness caused by epistasis, where residues interact non-linearly. Conventional evolutionary algorithms often struggle here: random multi-site mutations pose a high risk of destabilizing the native structure (low validity), while step-wise single-point strategies often fail to cross fitness valleys to reach higher peaks (local optima entrapment). Consequently, existing deep learning methods often rely on heavy structural annotations or task-specific training to mitigate this blindness, limiting their generalizability. This highlights an urgent need for a method that can heuristically guide multi-site exploration using intrinsic evolutionary priors, capturing residue dependencies without explicit structural supervision.

Protein language models (ProtLMs), build on Transformer architectures [29], have emerged as revolutionary tools by encoding evolutionary constraints from vast datasets (e.g., UniRef90 [30]). State-of-the-art ProtLMs (e.g., ESM-2 [31], ProtT5 [32]) capture epistasis relationships and co-evolutionary dependencies by masking specific positions and inferring likelihoods of all 20 amino acids. In structure-based design, continuous diffusion models such as RFdiffusion [33] and flow-matching techniques have revolutionized de novo backbone generation. These are typically coupled with powerful inverse folding networks like ProteinMPNN [34] to decode sequences from the generated 3D structures. In the sequence-only domain, discrete diffusion models like EvoDiff [35] operate directly in the sequence space, enabling the generation of diverse proteins without structural priors. Concurrently, reinforcement learning (RL) has been increasingly employed to fine-tune pre-trained PLMs, aligning sequence generation with specific functional rewards to navigate complex fitness landscapes [36].

Different with traditional evolutionary algorithms (e.g. GAs and MCTS) that rely on random mutagenesis, ProtLMs offer a principled approach to constrain combinatorial search spaces by heuristically inferring substitutions. This capability provides the foundation for more guided and evolutionarily consistent optimization strategies. Furthermore, the ability of ProtLMs to capture deep semantic patterns enables the design of multi-site mutations that are often inaccessible to traditional methods. By implicitly modeling the epistatic interactions between residues, ProtLMs provide a robust fitness proxy that correlates well with structural stability and evolutionary plausibility. This allows optimization strategies to confidently explore combinatorial spaces, identifying synergistic substitutions that enhance specific traits while maintaining the integrity of the protein fold, thus streamlining the path to improved variants.

**Fig 1:**
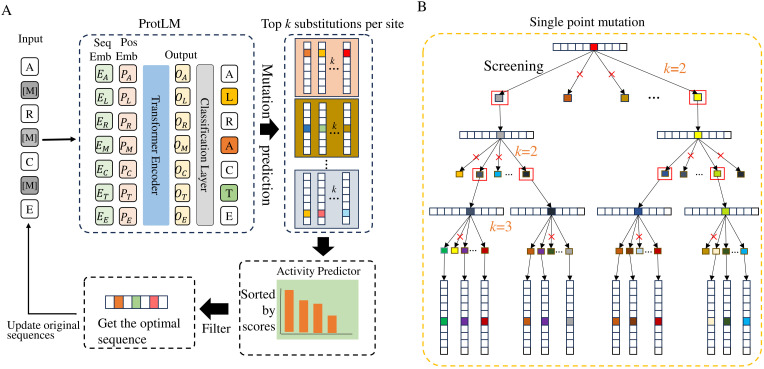
Illustration of heuristic multi-site optimization for protein sequence design using masked protein language models. **(A)** The iterative process of multi-site mutagenesis using ProtHMSO. **(B)** A illustration of narrowing exploration space via single-site mutation.

In this study, we proposed ProtHMSO, a heuristic multi-site optimization framework that leverages masked ProtLMs for protein sequence design. ProtHMSO employed substitution probability predicted by ProtLMs to heuristically guide the mutation selection, constraining combinatorial search spaces through evolutionary priors. Mutant sequences were iteratively refined through fitness evaluation, with top candidates updating the training set for subsequent optimization cycles. Through a dynamic masked language modeling mechanism, ProtHMSO implicitly captures epistasis and co-evolutionary information, updating substitution probabilities in real-time as the sequence context evolves without requiring any task-specific training. The framework of ProtHMSO is illustrated in **Fig 1**. ProtHMSO was also integrated as a heuristic mutation operator into GA and MCTS frameworks, enabling more effective navigation of their fitness landscape. Experimental results demonstrated its clear superiority in generating sequences that are both functionally optimized and naturally aligned. Our contributions are: **(1) A novel heuristic framework:** ProtHMSO leverages masked ProtLM predictions to prioritize substitutions that preserve structural integrity and function, narrowing the exploration space for sequence optimization. **(2) Efficient directed-optimization:** Iterative fitness evaluation and sequence refinement enable rapid convergence to high-performance variants. **(3) A modular plugin:** ProtHMSO enhances GA and MCTS efficiency as a modular plugin, demonstrating broad applicability in protein engineering.

## Preliminary knowledge and related work

### Protein Language Models

Protein language models [37], built upon Transformer encoders and multi-head self-attention mechanisms, effectively capture sequence contextual information via masked prediction tasks, facilitating broad applications in structure and function prediction. The pre-trained ESM-2 model [31] employs an improved BERT architecture with rotary position embeddings (RoPE) and scales up to 15 billion parameters. By eliminating the reliance on multiple sequence alignment (MSA) typical of traditional methods, ESM-2 efficiently infers atomic-level 3D structures directly from amino acid sequences, achieving inference speeds up to 60 times faster than technologies such as AlphaFold [38].

Leveraging its robust representation capabilities, ESM-2 excels in sequence mutation optimization tasks; for instance, its deployment within the MLDE framework [39] has demonstrated superiority over existing state-of-the-art methods. Inspired by this success, our ProtHMSO framework similarly utilizes ESM-2 for sequence mutation prediction, integrated with a pre-trained fitness scorer to enable direct, non-iterative optimization. This approach not only validates the efficacy of ESM-2 in mutation generation but also efficiently attains optimal results by circumventing the need for iterative processes.

### Genetic algorithm

The Genetic Algorithm (GA) is an efficient and parallel global search method inspired by biological evolution. Its operation relies on two fundamental mechanisms: **Natural Selection**, which determines reproductive eligibility based on fitness evaluations, ensuring that higher-quality solutions are prioritized; and **Genetics**, which utilizes crossover and mutation operations to recombine genetic material and introduce random alterations. These mechanisms allow the algorithm to maintain population diversity and autonomously explore the solution space to identify optimal results.

In the context of protein sequence optimization, GAs initialize a population from a sequence dataset and iteratively evolve offspring through hybridization and mutation, guided by fitness functions. While integrating machine learning with GAs has proven effective for optimizing molecules with desired biological activities [18,40,41], such methods typically demand large datasets and precise parameter tuning. To address these limitations, we embed the ProtHMSO as a heuristic operator within the GA architecture. This integration significantly enhances the algorithm’s ability to generate high-performance sequences without the heavy reliance on extensive initial libraries.

### Monte Carlo tree search

Monte Carlo Tree Search (MCTS) [42] is a heuristic strategy that optimizes decision-making through four key phases: selection, expansion, simulation, and backpropagation. To effectively balance exploration and exploitation during the selection phase, the algorithm typically employs the Upper Confidence Bounds (UCB) [43,44] formula:


$$\begin{array}{c} \text{UCB}(S_{i})={\overset{―}{V}}_{i}+c\sqrt{\frac{\log N}{n_{i}}} \end{array}$$ (1)

where ${\overset{―}{V}}_{i}$ represents the average estimated value of node *i*, *c* is the exploration constant (typically set to 2), *N* is the total number of visits to the parent node, and *n*_*i*_ is the visit count of node *i*. By weighing the estimated return against visit frequency, the UCB metric guides the search toward potentially high-quality paths until an unexplored or terminal node is reached.

In the expansion step, if the currently selected node is not a terminal node, one or more child nodes are created, representing possible next actions. These newly created child nodes are unexplored, so a random child node is selected to simulate the process until the game ends or a predetermined simulation depth is reached. The results of the simulation are then back-propagated along the selected path, updating the statistics of the nodes along the way, such as the number of visits. MCTS has been successfully applied to the optimization of protein sequences, as demonstrated by the EvoPlay framework proposed by Wang et al. [20]. This self-play reinforcement learning framework, based on the single-player version of AlphaZero [45], interacts with a policy-value neural network and a look-ahead MCTS to optimize sequences with single-point mutations, guided by both breadth and depth. In our work, we integrated ProtHMSO as a component within MCTS to enhance its effectiveness.

## Methodology

In this section, we present ProtHMSO, a heuristic multi-site optimization strategy. First, we introduced the core algorithm of ProtHMSO, which integrates a masked ProtLM to predict beneficial mutations as the heuristic search strategy. Then, we show how ProtHMSO can function not only as a standalone framework but also as a plug-and-play module that improves the convergence of genetic algorithms and Monte Carlo tree search.

The goal of directed evolution is to find the optimal sequence X^*^ that maximizes a particular objective function:


$$\begin{array}{c} X^{*}=\text{arg}\max_{X\in 𝒳}f(X) \end{array}$$ (2)

where 𝒳 represents the mutation space of sequences, and *f*(*X*) is the fitness function of sequence *X* in 𝒳. During optimization, the algorithm selects the best mutant sequence from those generated by mutations such that the returned fitness $y^{*}=f(X^{*})$ is the optimal fitness among all *y* values.

**Algorithm 1 Heuristic Multi-site Optimization Framework**

**Input:** Protein sequence $X=\{x_{1},x_{2},\ldots ,x_{n}\}$, and masked positions for mutation $M=\{m_{1},m_{2},\ldots ,m_{\left|M\right|}\}$:

**Output:** Optimized protein sequence

1:  N←∅

2:  **for** $(m_{i},m_{j},\cdots )\in M$ **do**       ▷ Select sites to be mutated

3:    $X^{\prime }\leftarrow \text{mask}(X,(m_{i},m_{j},\cdots ))$         ▷ Mask mutation sites

4:    $R\leftarrow \text{ESM2}(X^{\prime })$           ▷ Get substitution probabilities for masked sites

5:    C←sort and select top k from R

6:    **for** c∈C **do**

7:       $X_{c}\leftarrow \text{replace}(X,c)$               ▷ Create protein variants

8:       $score_{c}\leftarrow \text{score}(X_{c})$               ▷ Evaluate fitness

9:    **end for**

10:   $X_{max}\leftarrow \text{arg}\max_{X_{c}}(score_{c})$

11:   $N\leftarrow N\cup \{X_{\text{max}}\}$               ▷ Select the sequence with the highest score

12: **end for**                 ▷ Traverse all site combinations

13:  **return** $\text{arg}\max_{X_{c}}(N)$

### Heuristic Multi-site Optimization Framework

Heuristic multi-site optimization (HMSO) framework leverages the predicted beneficial mutations by ProtLMs for context-aware sequence exploration. The core mechanism of ProtLMs is masked language modeling (MLM), which is an unsupervised pretraining strategy by predicting masked tokens in varied contexts, therefore enabling dynamic context-aware mutation effects. Unlike static substitution matrices or existing methods that calculate site probabilities independently, our MLM-based strategy predicts substitutions for all masked sites synchronously based on global sequence context, implicitly capturing synergistic inter-residue epistasis zero-shot, which is the core unique mechanism of HMSO. Given a target sequence, a ProtLM is employed to predict the likelihood of substituting a specific residue with one of the 20 amino acids, thereby quantifying the impact of mutations on sequence fitness [46,47]. Specifically, a protein $X=(x_{1},\ldots ,x_{i},\ldots ,x_{L})$ has *L* residues, where the set of masked positions is $M=\{m_{1},\ldots ,m_{\left|M\right|}\}$. The masked residues can be inferred based on the surrounding contextual information. The probability of masked tokens is computed as $\prod_{m\in M}p(x_{m}|X_{⧵M})$, where $X_{⧵M}$ means all input tokens except those masked tokens.

Theoretically, the ability of ProtHMSO to capture synergistic inter-residue epistasis in a zero-shot manner is rooted in the multi-head self-attention mechanism of the Transformer architecture. During unsupervised pre-training on millions of evolutionary diverse sequences, the model learns the joint probability distribution of natural proteins. Because the self-attention mechanism computes pairwise attention scores between all tokens regardless of their sequence distance, the conditional probability $p(x_{m}|X_{⧵M})$ dynamically encodes global structural and co-evolutionary dependencies. Consequently, the mutational effect at a specific site is not evaluated in isolation; rather, it is strictly conditioned on the genetic background of the entire sequence. If a residue at site *j* is altered, the contextual embedding and the resulting substitution probability at site *i* will shift accordingly. This intrinsic mathematical property allows the model to implicitly quantify non-linear epistatic interactions between residues without relying on explicit structural data or task-specific supervised training. In this study, ESM-2 [31] was primarily used as the masked ProtLMs to predict mutation sites of input sequences. The process of the HMSO framework is illustrated in **Fig 1**.

The core process of our proposed HMSO is described in **Algorithm 1**. HMSO takes a protein sequence *X* and the corresponding mutation sites *M* as inputs, and outputs the optimized sequence *N*. The protein sequence *X* is first masked according to the selected mutation sites and then fed into the ESM-2 model to get their substitution probabilities of 20 natural amino acids at each mutation site. These probabilities are then sorted in descending order, the top *k* substitutions are selected, and the corresponding *k* protein variants are generated. Then these *k* variants are assigned a score by a fitness evaluator, subsequently the sequence with the highest score in each mutation is selected and added to the final set of mutant sequences. This process is iterated to eventually obtain an optimized sequence. Experiments have shown that a value of *k* of 3 yields the best overall results. For detailed experimental information, please refer to Supplementary Information S.1 in S1 File. Furthermore, unlike most standalone PLM-based methods, HMSO’s core heuristic mutation strategy can be directly integrated into classic optimization frameworks (such as genetic algorithms and Monte Carlo tree search) to replace blind random mutation, thereby greatly expanding its application scope in protein engineering.

### Heuristic Genetic Algorithm based on HMSO

**Fig 2:**
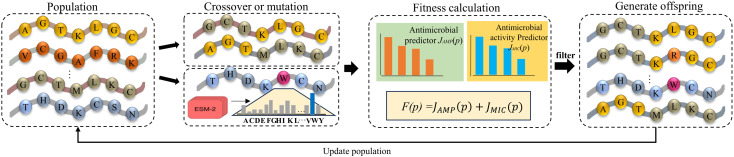
Improved genetic algorithm framework using ESM-2. The protein sequence is used to initialize the population. In the mutation operation, the ESM-2 model is used instead of random mutation. The weighted scores of each predictor and the custom error term are used as the fitness score to screen the offspring and update the population.

HMSO is not only a standalone framework but also a modular plugin that enhances the convergence efficiency of genetic algorithms by guiding exploration with evolutionary priors. In traditional GAs, random crossover/mutation operators lack biological plausibility, often disrupt conserved structural motifs, and single-site optimization neglects epistatic interactions between distant residues. To overcome these challenges, we proposed GA-HMSO, a heuristic genetic algorithm based on HMSO, by employing HMSO to heuristically guide GA mutation operation, enabling it to address the limitations of conventional random mutation and low-diversity offspring generation. First, we initialize the population with the prepared protein sequence, and then select mutation and crossover operations based on probability. The crossover process uses two-point crossover, and the mutation process uses ESM-2 to guide mutation. After completing the crossover mutation operation, the generated sequence is evaluated, and then the offspring is screened according to the fitness function to update the parent generation. This heuristic framework enables simultaneous optimization of multiple sites, leveraging the ProtLMs ability to capture long-range interactions and evolutionary constraints, which is particularly critical for navigating complex fitness landscapes in protein design. At the same time, we also use dynamic crossover mutation probability to prevent it from falling into the local optimal solution problem. The framework of GA-HMSO is illustrated in **Fig 2**.

**Algorithm 2 Heuristic Genetic Algorithm based on HMSO**

**Input:** Protein sequence dataset *S*, population size *P*, number of iterations *N*, threshold *g*

**Output:** Optimized protein sequence dataset M

1:  **Initialize** population by selecting *P* sequences from dataset *S*

2:  **for** gen←1 to **N do**

3:    **if** *gen* ≤ *g* **then**

4:     mutation_rate←high_rate

5:   **else**

6:      mutation_rate←low_rate

7:   **end if**

8:   **select** *operation* based on mutation_rate and crossover_rate

9:   **if** *operation* is crossover **then**

10:    **for** each selected pair of parents *x*_*i*_ and *x*_*j*_ **do**

11:     Perform crossover to generate new offspring**:**

12:     $x_{\text{new}}=(x_{i_{1}},x_{i_{2}},\ldots ,x_{i_{c}},x_{j_{\left(c+1\right)}},\ldots ,x_{j_{n}})$

13:    **end for**

14:   **else**

15:    **Use** ESM-2 model to predict mutations for the sequence and apply the mutations to generate the mutated sequence.

16:   **end if**

17:   **evaluate** fitness for each individual in population

$f(x)=J_{\text{1}}(x)+J_{\text{2}}(x)+...+J_{\text{n}}(x)$

18:  **select** individuals for next generation based on fitness

$p_{i}=\frac{f(x_{i})}{\sum_{j=1}^{P}f(x_{j})}$

19:  **end for**

20:  **return** *M*

An individual being selected into the next population is depending on its fitness. Let *p*_*i*_ be the probability of the *i*-th individual being selected, then:


$$\begin{array}{c} p_{i}=\frac{f(x_{i})}{\sum_{j=1}^{N}f(x_{j})} \end{array}$$ (3)

where *f*(*x*_*i*_) represents the fitness of the *i*-th individual, and *N* denotes the number of individuals in the generated offspring. The definition of the fitness function *f*(*x*) is a combination of multiple attributes, as follows:


$$\begin{array}{c} f(x)=J_{\text{1}}(x)+J_{\text{2}}(x)+...+J_{\text{n}}(x) \end{array}$$ (4)

where *J*_i_(*x*) represents the attribute score of the protein sequence. The scores of these predictors are added together as the final score. Therefore, GA-HMSO can be employed for multiple objective optimization. Selected individuals are then combined through a crossover operation to generate new offspring. Assuming the crossover point is *c*, and considering two individuals *x*_*i*_ and *x*_*j*_, the offspring *x*_new_ can be represented as $x_{\text{new}}=(x_{i_{1}},x_{i_{2}},\ldots ,x_{i_{c}},x_{j_{\left(c+1\right)}},\ldots ,x_{j_{n}})$.

During the selection phase, we use the ’[mask]’ token to replace the amino acids at selected positions of the sequences, represented by $x_{m}=(x_{1},x_{2},\cdots ,[mask],\cdots ,[mask],\cdots ,x_{n})$. This mask vector allows for the selective and dynamic combination of sequence components, thereby enhancing the algorithm’s efficiency in optimization. In the early iterations, we set a high mutation probability to promote broad exploration of the vast sequence space, preventing the population from prematurely converging to local optima. As the number of iterations increases, the algorithm shifts its focus toward exploitation; the mutation probability dynamically decreases to preserve high-fitness structural motifs (schemas) discovered in earlier generations, thereby refining the local optimization. For details on the choice of probability and the comparison of algebraic thresholds, please refer to the Supplementary Information S.3 in S1 File. Ultimately, we chose dynamic probability in this experiment. Specifically, the mutation probability *P*_MUT_ and the crossover probability *P*_CX_ which were manually chosen vary according to the generation number *gen* as follows:


$$\begin{array}{c} P_{\text{MUT}}=\left\{ \begin{array}{cc} 0.7 & \text{if}\;\text{gen}\leq g\\ 0.5 & \text{if}\;\text{gen}>g \end{array}\right.\;P_{\text{CX}}=\left\{ \begin{array}{cc} 0.5 & \text{if}\;\text{gen}\leq g\\ 0.4 & \text{if}\;\text{gen}>g \end{array}\right. \end{array}$$ (5)

where *P*_MUT_ represents the mutation probability, and *P*_CX_ represents the crossover probability, with *g* being the generation threshold after which the probabilities are adjusted. This adjustment is due to the fact that a higher mutation probability enhances global optimization, but after a certain number of generations, the population tends to converge to local extrema. At this point, with a large number of mutations, the mutation probability is reduced in the later stages to focus more on local optimization. Finally, offspring are selected based on their fitness scores, and the selected offspring are used to update the population for the next iteration. The process of GA-HMSO is described in **Algorithm 2**.

### Heuristic Monte Carlo Tree Search based on HMSO

**Fig 3:**
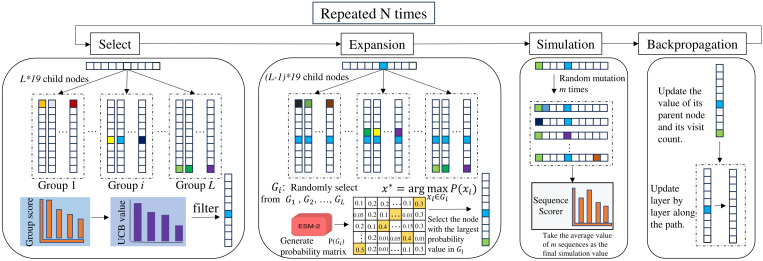
Heuristic Monte Carlo Tree Search based on HMSO. First, the subnode to be expanded is selected according to the UCB value, and then the top *k* subnodes are generated for each site according to the probability matrix generated by ESM-2. Finally, one of the subnodes is randomly returned. After simulating the subnodes, they are updated layer by layer along the generation path of the subnodes.

Traditional Monte Carlo Tree Search relies on random simulation and node selection strategies based on UCB formula. It is difficult to efficiently explore potential mutation combinations in the high-dimensional space of protein sequence design. In its expansion step, the number of child nodes generated by each node grows exponentially with the length of the sequence, resulting in a shallow search tree, a large number of valuable mutation paths being ignored, and falling into a local optimal solution. For example, in the design of antimicrobial peptides, traditional MCTS requires thousands of iterations to converge, and often generates invalid sequences due to random mutations, wasting computing resources. To address this, in this study, we proposed MCTS-HMSO by combining MCTS with HMSO to heuristically optimize protein sequences. The framework is shown in **Fig 3**.

**Algorithm 3 Heuristic Monte Carlo Tree Search based on HMSO**

**Input:** Protein sequence *X*, number of iterations *N*

**Output:** Optimized protein sequence X’

1: Create root node for sequence *X*

2:  **for** each iteration t = 1 to *N* **do**

3:   **Selection Step:**

4:    Select the group with the highest score *S*(*G*_best_)

5:    Select the child node with the highest UCB value within group *G*_best_:

$j^{*}=\underset{j\in \text{arg}\max_{i}S(G_{i})}{\text{arg}\max}{\text{UCB}}_{i,j}$

6:    Update the score of the selected group *G*_best_ by summing the UCB values of its nodes

7:   **Expansion Step:**

8:    Generate probability matrix *P* for *S* using ESM-2.

9:    **for** a∈unmutated sites M **do**

10:     Add a new child $x^{\prime }$ to *x.child*

11:      $x^{\prime }\leftarrow P(x^{\prime })$

12:    **end for**

13:    $G_{\text{selected}}=\text{Randomly select from }\{G_{1},\ldots ,G_{m}\}$

14:    **Return:** $x^{*}=\text{arg}\max_{x_{i}\in G_{\text{selected}}}P(x_{i})$

15:   **Simulation Step:**

16:    Mutate the sequence multiple times and average the results for the final simulated value.

$\overset{―}{f}=\frac{1}{M}\sum_{i=1}^{M}f(x_{i})$

17:   **Backtracking Step:**

18:    Update child node information along mutation path.

19:  **end for**

20:  **Return:** The optimized protein sequence $X^{\prime }$

In MCTS-HMSO, a node represents a protein sequence. The root node is the original protein sequence to be optimized. The structure of the entire tree is as follows: the first layer contains the root node, and the second layer contains 19 × *L* child nodes, each representing one of the 19 × *L* possible mutated sequences. When the child nodes of the second layer are expanded, each generates 19×(L−1) child nodes, meaning that previously mutated positions will no longer undergo mutation in subsequent layers. Instead, mutations will occur at different positions. Generally, the number of child nodes generated at the *i* -th layer is 19×(L−i−1) (with the root node being the first layer).

During the expansion step, to heuristically explore these potential protein variants, all sub-nodes are grouped into *L* groups according to their mutation positions. The underlying philosophy behind employing group-based UCB values is to mitigate the severe combinatorial explosion inherent in multi-site protein design. Standard MCTS treats every single amino acid substitution as an independent action, which leads to an excessively wide and shallow search tree. By grouping sub-nodes based on their mutation positions, we introduce a two-step decision-making process. The algorithm first identifies which specific residue site is most critical to mutate (macro-exploration driven by the group score), and subsequently selects the most promising amino acid substitution within that site (micro-exploitation driven by individual UCB values). This hierarchical pruning focuses the computational budget on the most impactful structural regions. Then, each sub-node is assigned a substitution score by employing ESM-2. Each group is also assigned a score by summing the UCB values of all nodes within the group. The initial score of each group is set to infinity, and the initial UCB value of each sub-node is also infinite.

In the selection process, the group is first selected based on the score of each group, and then the sub-node with the highest UCB value is chosen within the selected group. Specifically, given a group *G*_*i*_ consisting of nodes $x_{1},x_{2},\ldots ,x_{n}$, where each node *x*_*j*_ has a UCB value *UCB*(*x*_*j*_). In the Supplementary Information S.4 in S1 File, we compared the scores of the groups by selecting the maximum value of the explored nodes as the group score, and the final performance of both methods was comparable. In this experiment, we chose the weighted sum of the explored nodes as the group score. The nodes that have been explored are $x_{1},x_{2},\ldots ,x_{e}$ and the score of the group *S*(*G*_*i*_) can be represented as:


$$\begin{array}{c} S(G_{i})=\sum_{j=1}^{e}UCB(x_{j}) \end{array}$$ (6)

where *e* is the number of nodes explored in group *G*_*i*_, and the *UCB* value of each node is still calculated according to Eq. (1). After calculating the *UCB* value, the score of the group to which the current node belongs will be updated accordingly. Finally, the final child node is selected according to the following formula:


$$\begin{array}{c} j^{*}=\underset{j\in \text{arg}\max_{i}S(G_{i})}{\text{arg}\max}{\text{UCB}}_{i,j} \end{array}$$ (7)

This sequence is then used for the next simulation step. In our experiment, we perform a series of random mutations on the selected sequence, and then evaluate the fitness of mutated variants, at last take the average of fitness scores as the final simulated value for the selected sequence. During the backtracking process, both the sub-node score and its group score need to be updated. The algorithm of MCTS-HMSO is outlined in **Algorithm 3**.

### Experiments

To evaluate the efficacy of ProtHMSO, we conducted comparative analyses against random mutation strategies, conventional GA and conventional MCTS. These comparisons aimed to assess both the stand-alone performance of ProtHMSO and its integration as a heuristic component in GA (GA-HMSO) and MCTS (MCTS-HMSO). Experiments were performed on two benchmark datasets: an AMP dataset and the ProteinGym database, enabling comprehensive evaluation across diverse optimization scenarios.

### Masked protein language models

We employed ESM-2 (650M), as the masked ProtLMs, to heuristically guide mutation. The ESM-2 architecture has 650M parameters, including 33 transformer layers with a maximum sequence length of 1,024 tokens, a hidden dimension of 1,280 and 20 attention heads. Its vocabulary includes 25 amino acids, including 5 non-natural residues, while the predictor can only recognize the 20 natural amino acids. To ensure biological relevance, predictions corresponding to non-natural amino acids were substituted with lysine (K), following the protocol established by Zhang et al. [19]. All optimizations were performed using single-precision floating-point arithmetic on NVIDIA A100 GPUs.

### Antimicrobial Peptide dataset

All antimicrobial peptides were sourced from the Database of Antimicrobial Activity and Structure of Peptides (DBAASP) [48], which catalogs peptides along with activity values and known hemolytic behaviors. From the initial dataset comprising 11,805 non-cyclic peptides, we retained 4,505 unique active sequences after deduplication, following the preprocessing by Capecchi et al. [49]. To comprehensively evaluate the algorithm’s capacity to improve peptide performance under varying initial conditions, we followed the dataset partitioning strategy described by Liu et al. [50]. Furthermore, to facilitate the generation of peptides with enhanced activity, we increased the probability threshold *P*_MIC_ to 0.8. The specific breakdown is as follows: **Case 0: High-active AMPs** ($P_{\text{AMP}}\geq 0.8,P_{\text{MIC}}\geq 0.8$). These sequences already exhibit optimal antimicrobial properties and do not require further optimization. **Case 1: Activity-enhancement candidates** ($P_{\text{AMP}}\geq 0.8,P_{\text{MIC}}\leq 0.8$). Optimization targets improvement of antimicrobial activity while preserving functionality. **Case 2: Functionality-enhancement candidates** ($P_{\text{AMP}}\leq 0.8,P_{\text{MIC}}\geq 0.8$). Optimization focuses on enhancing the antimicrobial function. **Case 3: High-challenge sequences** ($P_{\text{AMP}}\leq 0.8,P_{\text{MIC}}\leq 0.8$). These sequences represent the most challenging optimization scenario and demand optimization of both functionality and activity. There are 875, 184, 476 peptide sequences in Case 1, Case2 and Case3.

### ProteinGym datasets

ProteinGym [51] is a collection of benchmarks aiming at comparing the ability of models to predict the effects of protein mutations. The benchmarks in ProteinGym were divided according to mutation type (substitutions vs. indels) and ground truth source (DMS assay vs. clinical annotation). In this study, we focused on the substitution type, where DMS benchmark contains 217 assays spanning over 2.4 million mutants and clinical benchmark contains 2,525 genes spanning over 63 thousand mutants. The mutant fitness of DMS substitutions were evaluated by DMS_score, which is a continuous experimental measurement. Higher values indicate high-fitness, while the mutant fitness of clinical substitutions were evaluated by DMS_score_bin, which is a binary label indicating whether the DMS_score exceeds a fitness threshold (1: pathogenic, 0: benign).

To further investigate site-specific optimization, we conducted targeted mutagenesis at functional residues. Active sites were first identified using NCBI’s Conserved Domain Database (CDD) and literature annotations. Mutants were then introduced at these positions via both random and ProtHMSO methods. The resulting mutants were assessed by comparing their DMS_score distributions to quantify performance differences between the two strategies.

### Metrics

To holistically evaluate our heuristic optimization framework, we employed four complementary metrics spanning functional and structural assessments.

For the AMP optimization, two functional fitness metrics: antimicrobial probability ($P_{\text{AMP}}\in [0,1]$) and minimal inhibitory concentration probability ($P_{\text{MIC}}\in [0,1]$) were employed to quantify the likelihood of a sequence being antimicrobial peptide and retaining effective antimicrobial activity against gut microbiota at low concentrations (≤32μg/mL). These indicators were predicted by the classification model derived from HydrAMP [52]. Furthermore, to evaluate the robustness of our method, we assessed the impact of noise on the predictor’s evaluation performance in Supplementary Information S.6 in S1 File.

For the evaluation of structural validity and biological plausibility of designed sequences, we utilized two additional indicators: predicted local Distance Difference Test (pLDDT) scores and perplexity. pLDDT scores reflect the confidence in predicted backbone atom positions with higher values indicating greater structural validity. In this study, pLDDT scores were computed via ESMFold [31]. Perplexity evaluates the negative log-likelihood of sequences under a generative protein language model. Lower perplexity values indicate closer alignment with natural sequence distributions, correlating with higher experimental expressibility. In this study, perplexity is calculated via ProGen2 [53]. To further verify the biophysical properties of the generated variants, we evaluated the energy values of the generated variants in Supplementary Information S.7 in S1 File.

### Results and discussion

We comprehensively evaluate ProtHMSO across multiple experimental configurations, benchmarking its efficacy with random mutation strategies, and then assessing its integration with GAs and MCTS. The time efficiency comparison of the various methods and the rationale for using ProtLM are included in the Supplementary Information S.2 and S.5 in S1 File.

### Improving functionality and activity of antimicrobial peptides via ProtHMSO

We first compared ProtHMSO with random mutagenesis on the AMP dataset. Random mutation is a mutation with equal probability at each site. Since peptides are short amino acid sequences with relative same mutation space, we could conduct multiple mutations simultaneously. Mutants were performed across 1–5 variable sites per sequence. Due to the vast compositions, we limited the mutation times to reduce exploration space. For single-point mutations, each site of the sequence was mutated once across the sequence length, and the variant with highest score was selected as the final optimized sequence. For multiple-point mutagenesis, the mutated sites in each iteration were first randomly selected, and then all substitutions were determined simultaneously via random mutagenesis or ProtHMSO, respectively. The number of mutants is the length of the corresponding sequence. The variant with the highest score is selected as the final optimized sequence of the corresponding mutation method.

**Fig 4:**
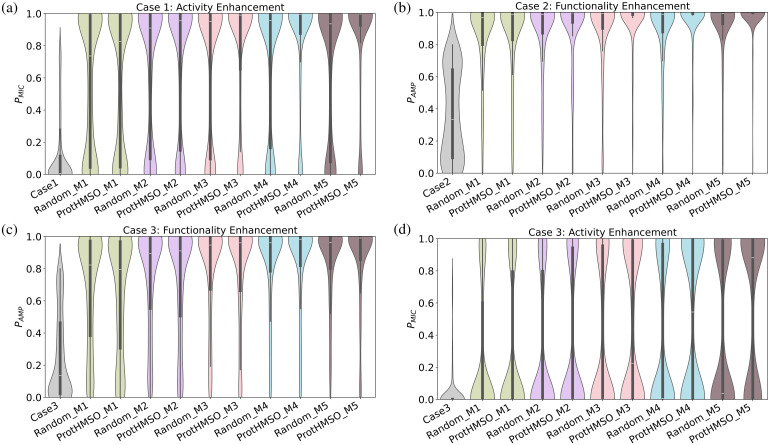
Comparison of functionality or activity enhancement of three AMP cases via random and ProtHMSO guided mutagenesis. **(a)** The activity-enhancement of AMPs in Case1. **(b)** The functionality-enhancement of AMPs in Case2. **(c-d)** The functionality-enhancement (c) and activity-enhancement (d) of AMPs in Case3. *Case1*, *Case2* and *Case3* represent the distribution of AMP cases. *Random_M1* represents single-site mutation using a random method, and so on.

The evaluation of optimized sequences on the three cases in AMP dataset in terms of the average *P*_*AMP*_ and *P*_*MIC*_ (**Table 1**) and their distributions (**Fig 4**) showed that both random and ProtHMSO methods enhanced the activity and functionality of all three cases, compared with the original sequences across 1–5 sites mutation. Moreover, ProtHMSO consistently performed better than random method, where ProtHMSO achieved the highest average *P*_*MIC*_ of 0.82 for Case1, *P*_*AMP*_ of 0.96 for Case2, and *P*_*AMP*_ of 0.86 and *P*_*MIC*_ of 0.56 for Case3. These results demonstrated ProtHMSO can improve functionality and activity of AMPs by heuristically searching, even for the high-challenge candidates that demand optimization of both functionality and activity.

**Table 1:** Comparison the average performance of sequences generated using different mutation methods in terms of *P*_*AMP*_, *P*_*MIC*_, pLDDT and perplexity across three AMP cases.

Methods	Case1			Case2			Case3			
	$P_{MIC}↑$	pLDDT↑	perplexity↓	$P_{AMP}↑$	pLDDT↑	perplexity↓	$P_{AMP}↑$	$P_{MIC}↑$	pLDDT↑	perplexity↓
Random_M1	0.57(±0.42)	70.51(±11.68)	17.45(±5.23)	0.85(±0.24)	70.82(±8.39)	17.65(±3.53)	0.68(±0.34)	0.26(±0.39)	67.89(±11.55)	17.21(±4.59)
Random_M2	0.63(±0.42)	70.01(±11.45)	18.21(±4.91)	0.87(±0.22)	70.81(±9.00)	18.16(±3.55)	0.74(±0.32)	0.30(0.41)	67.69(±11.33)	17.85(±4.58)
Random_M3	0.66(±0.43)	69.34(±11.26)	18.72(±4.95)	0.89(±0.23)	70.75(±8.58)	18.85(±4.69)	0.78(±0.30)	0.37(±0.45)	67.09(±11.19)	18.37(±4.51)
Random_M4	0.67(±0.42)	68.56(±11.16)	19.33(±4.71)	0.89(±0.14)	70.12(±9.11)	19.22(±3.84)	0.82(±0.27)	0.38(±0.45)	66.72(±11.49)	18.70(±3.91)
Random_M5	0.65(±0.43)	67.67(±11.00)	19.85(±4.54)	0.91(±0.20)	70.02(±8.71)	19.63(±3.31)	0.84(±0.25)	0.42(±0.46)	65.98(±11.05)	19.39(±4.20)
ProtHMSO_M1	0.58(±0.42)	71.90(±11.82)	15.93(±4.66)	0.85(±0.25)	73.05(±8.21)	16.23(±3.07)	0.64(±0.36)	0.28(±0.41)	69.73(±11.46)	16.08(±4.29)
ProtHMSO_M2	0.67(±0.41)	71.84(±11.76)	15.86(±4.35)	0.89(±0.22)	73.56(±8.73)	15.99(±3.15)	0.72(±0.34)	0.34(±0.44)	69.76(±11.52)	16.07(±3.76)
ProtHMSO_M3	0.76(±0.37)	71.75(±11.41)	15.66(±4.09)	0.93(±0.18)	73.92(±8.17)	15.56(±2.83)	0.79(±0.31)	0.45(±0.46)	70.24(±11.49)	15.80(±3.66)
ProtHMSO_M4	0.81(±0.33)	71.73(±11.58)	15.48(±4.69)	0.95(±0.16)	73.85(±8.91)	15.17(±2.79)	0.83(±0.28)	0.49(±0.46)	71.15(±11.22)	15.60(±3.37)
ProtHMSO_M5	**0.82** (±0.33)	**71.56** (±11.46)	**14.82** (±3.74)	**0.96** (±0.13)	**74.76** (±8.08)	**14.43** (±2.68)	**0.86** (±0.26)	**0.56** (±0.46)	**71.25** (±11.26)	**15.13** (±3.24)
GA	0.09(±0.28)	62.62(±9.92)	18.96(±3.04)	0.31(±0.41)	66.50(±8.11)	19.53(±2.78)	0.43(±0.37)	0.06(±0.20)	59.88(±9.18)	19.19(±2.10)
GA-HMSO_fixed	0.43(±0.46)	69.41(±10.94)	12.17(±3.48)	0.88(±0.25)	74.68(±8.34)	9.82(±4.32)	0.60(±0.42)	0.29(±0.42)	67.98(±11.00)	12.93(±3.44)
GA-HMSO_dynamic	**0.75** (±0.41)	**81.62** (±9.18)	**5.20** (±2.85)	**0.90** (±0.29)	**80.87** (±9.12)	**5.82** (±2.85)	**0.90** (±0.25)	**0.83** (±0.34)	**82.37** (±9.56)	**4.52** (±2.56)
MCTS_100	0.73(±0.40)	70.80(±11.50)	17.68(±5.43)	0.94(±0.14)	71.74(±8.46)	17.97(±3.61)	0.76(±0.31)	0.34(±0.44)	67.92(±11.59)	17.49(±4.40)
MCTS_300	0.81(±0.35)	70.66(±11.55)	18.06(±5.18)	0.96(±0.11)	71.05(±8.68)	17.95(±4.25)	0.82(±0.28)	0.42(±0.46)	68.21(±11.47)	17.62(±4.37)
MCTS_500	0.86(±0.31)	70.42(±11.73)	18.40(±5.80)	0.98(±0.08)	71.48(±8.75)	18.42(±5.08)	0.87(±0.24)	0.42(±0.47)	67.92(±11.56)	17.86(±5.04)
MCTS-HMSO_100	0.74(±0.40)	71.93(±11.45)	16.39(±4.63)	0.96(±0.13)	73.21(±8.39)	16.41(±3.06)	0.82(±0.30)	0.40(±0.46)	69.48(±11.36)	16.26(±4.01)
MCTS-HMSO_300	0.83(±0.35)	71.90(±11.43)	16.45(±4.45)	0.97(±0.12)	73.49(±8.17)	16.22(±3.07)	0.92(±0.21)	0.58(±0.47)	69.93(±11.50)	16.32(±3.92)
MCTS-HMSO_500	**0.88** (±0.29)	**72.13** (±11.46)	**16.35** (±4.39)	**0.98** (±0.09)	**73.51** (±9.00)	**16.32** (±3.12)	**0.96** (±0.13)	**0.66** (±0.45)	**69.93** (±11.54)	**16.31** (±3.98)

The bold numbers represent the best performance within each kind of method. The arrows (↑) indicate that higher values are better, while (↓) indicates lower values are better. Values in parentheses (±...) represents the standard deviation.

Besides, theoretically, as the number of mutated sites increases, more potential sequences with high functionality and activity could be explored, subsequently a larger improvement would be achieved. Interestingly, for Case1, random 5-site mutation led to lower activity improvement than 4-site, likely due to exponential sparsity of functional sequences in the combinatorial explosion of mutation space. In the same number of searching iterations, it is exponentially hard to find better variants. However, we observed that ProtHMSO achieved a higher average *P*_*MIC*_ in the combinatorial explosion of mutation space across all three cases. Notably, as the number of mutated sites increasing, the pLDDT and perplexity metrics achieved by ProtHMSO were also getting better, in contrast the performance of random method was getting worse, indicating that the sequences optimized by ProtHMSO retained high structural validity and sequential plausibility, while the random mutants would break these rationality. This divergence suggests ProtHMSO can leverage epistatic interactions to heuristically guide multi-site combinatorial improvements.

### Exploring beneficial mutation of long protein sequences via ProtHMSO

To rigorously evaluate the efficacy of ProtHMSO, we conducted long protein optimization on the ProteinGym database, where the average length of the Clinical benchmark is 709 and the average length of DMS benchmark is 397. Due to the vast composition, we only focused on the single-site substitutions.

**Fig 5:**
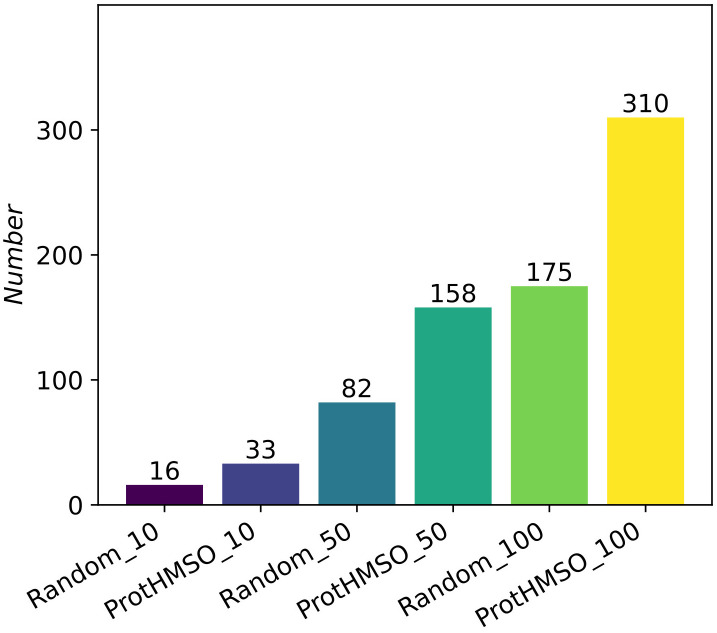
Number of non-pathogenic protein sequences optimized by random and ProtHMSO guided mutagenesis. *Random_10* means to generate 10 sequences using random mutation for each original sequence, and so on.

The Clinical benchmark comprises experimentally validated single-site substitutions associated with pathogenicity. We introduced single-site mutations at identical positions in the original sequences using both random mutagenesis and ProtHMSO guided approach. Each sequence generated 10, 50, and 100 mutated variants under each strategy. As shown in **Fig 5**, ProtHMSO significantly outperformed random mutagenesis, generating approximately twice as many non-pathogenic variants as random mutagenesis across all numbers of mutants. These results indicate that ProtHMSO leverages the evolutionary constraints encoded in the protein language model to prioritize functionally benign mutations while mitigating pathogenic outcomes.

**Fig 6:**
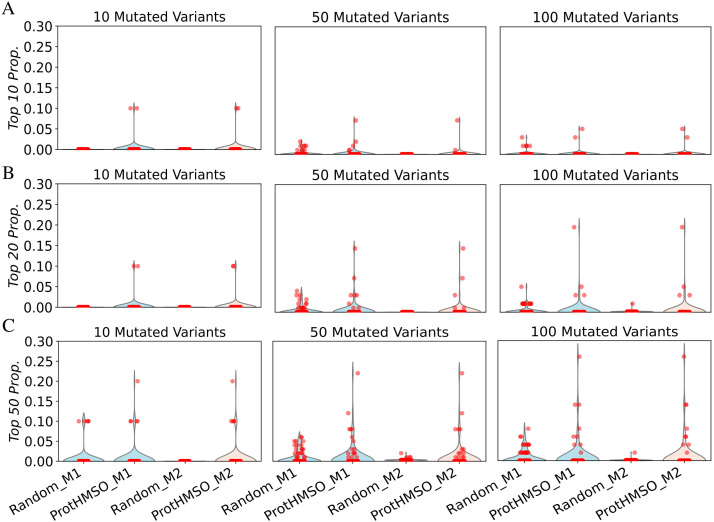
The proportion of mutated variants in top 10, 20 and 50 highest fitness scores. **A.** The proportion of the top 10 mutated variants with the highest scores among 10, 50, and 100 mutants generated using random mutation and ProtHMSO in the DMS dataset. **B.** The proportion of the top 20 mutated variants with the highest scores among 10, 50, and 100 mutants generated using random mutation and ProtHMSO in the DMS dataset. **C.** The proportion of the top 50 mutated variants with the highest scores among 10, 50, and 100 mutants generated using random mutation and ProtHMSO in the DMS dataset. *Random_M1* and *ProtHMSO_M2* respectively represent the use of random single-point mutation and the use of ProtHMSO to mutate two sites, and so on.

The DMS benchmark contains both single and two mutant variants with experimentally measured fitness scores (DMS_score). We partitioned this dataset into single-site and two-site mutation subsets, then ranking variants within each subset by their DMS_score. Functional sites for mutation were identified through conserved domain annotations from the NCBI database, which integrates structural and evolutionary analyses from the Conserved Domain Database [54,55]. For each sequence, we generated mutant libraries targeting these functional sites using both random mutagenesis and ProtHMSO. The top 10, 20 and 50 experimentally ranked mutants were selected as high-fitness benchmarks. We then assessed the overlap between generated mutants and these top experimental variants. As shown in **Fig 6**, PortHMSO consistently outperformed random mutagenesis across both single and two-site mutations. Notably, ProtHMSO-generated mutants exhibited significantly higher enrichment in top-10, 20 and 50 high-fitness variants. This systematic improvement underscores the advantage of evolutionary-aware language model guidance over stochastic exploration for identifying functional sequence variants.

### Accelerating genetic algorithm convergence via GA-HMSO

To evaluate the efficacy of integrating ProtHMSO as a component into GA framework, we conducted comparative analyses between conventional GA and two GA-HMSO variants: a fixed crossover mutation rate (GA-HMSO_fixed), and a dynamic crossover mutation rate (GA-HMSO_dynamic). GA-HMSO_fixed retained the default crossover mutation rate of conventional GA, while GA-HMSO_dynamic adopted an adaptive strategy, starting with a higher mutation rate to prioritize exploration and then gradually reducing to a lower rate for intensified exploitation. All algorithms were initialized with identical populations derived from three cases in AMP datasets.

**Fig 7:**
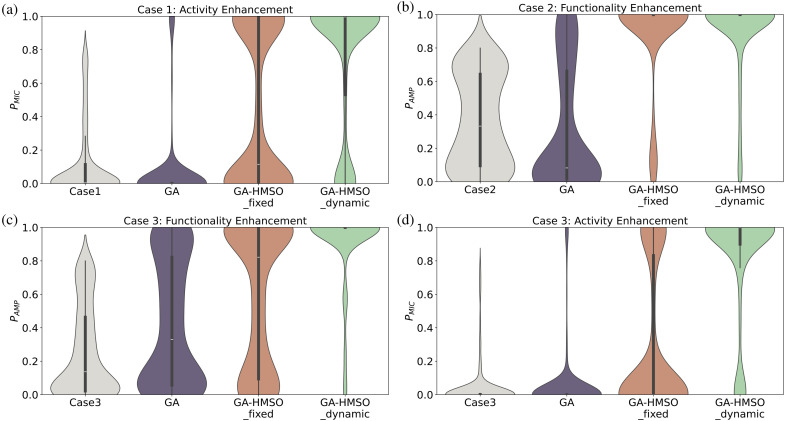
Comparison of functionality or activity enhancement of three AMP cases via GA and two GA-HMSO variants (GA-HMSO_fixed and GA-HMSO_dynamic). **(a)** The activity-enhancement of AMPs in Case1. **(b)** The functionality-enhancement of AMPs in Case2. **(c-d)** The functionality-enhancement (c) and activity-enhancement (d) of AMPs in Case3.

As shown in **Fig 7**, conventional GA successfully enhanced the functionality of Case2 and Case3, but failed to improve the activity of Case1 and Case3. In contrast, both GA-HMSO_fixed and GA-HMSO_dynamic demonstrated consistent superiority across all three cases compared to conventional GA, particularly evident in Case 3, which contains high challenge candidates with low *P*_AMP_ and *P*_MIC_. Notably, as shown in **Table 1**, GA-HMSO_dynamic achieved the highest performance with average *P*_*MIC*_ of 0.82 and 0.83 for Case1 and Case3, outperforming GA-HMSO_fixed with average *P*_*MIC*_ of 0.43 and 0.29 for Case1 and Case3. Besides, GA-HMSO_dynamic achieved all average pLDDT values higher than 80 and all average perplexity scores lower than 6 across three AMP cases, which were about 10% better than conventional GA and GA-HMSO-fixed. These results illustrated that by implementing an adaptive mutation schedule, GA-HMSO_dynamic effectively balanced global search breadth and local fitness landscape refinement, providing particularly advantageous for navigating the complex, high-dimensional fitness landscape of multi-site protein sequence optimization.

The performance differentials underscore the critical role of ProtHMSO operators derived from protein language models. By leveraging evolutionary principles alongside learned biological constraints, the hybrid GA-HMSO framework enables efficient exploration of combinatorial sequence space while preserving biological plausibility. This addresses fundamental limitations of conventional evolutionary strategies in computational protein design, particularly in scenarios requiring simultaneous optimization of multiple distant residues.

### Enhancing MCTS exploration via MCTS-HMSO

**Fig 8:**
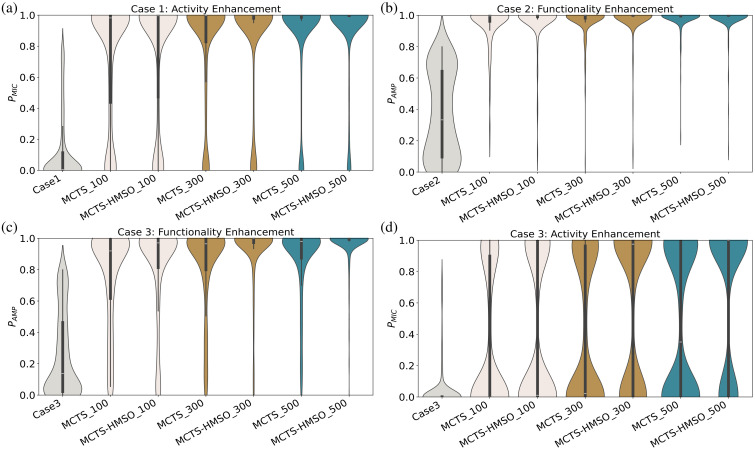
Comparison of functionality or activity enhancement of three AMP cases via MCTS and MCTS-HMSO. **(a)** The activity-enhancement of AMPs in Case1. **(b)** The functionality-enhancement of AMPs in Case2. **(c-d)** The functionality-enhancement (c) and activity-enhancement (d) of AMPs in Case3. *MCTS_100* means the sequence generated by MCTS at 100th iteration, and so on.

To evaluate the efficacy of integrating ProtHMSO as a component in MCTS framework, we conducted a comparison between conventional MCTS and its heuristic variant (MCTS-HMSO) across three AMP cases. The exploration efficiency of both methods was systematically analyzed by assessing their optimization outcomes at iterative checkpoints (100, 300, and 500 iterations), as shown in **Fig 8**.

Overall, both MCTS and MCTS-HMSO exhibited significant improvement in antimicrobial functionality and activity properties relative to original sequences, and the improvement was further enhanced as the iteration progressed. Moreover, although MCTS has achieved quite high performance, MCTS-HMSO consistently performed better than the MCTS at all three iterative checkpoints across three AMP cases. This superior performance underscores the enhanced exploration efficiency afforded by the HMSO operator, which leverages structural and evolutionary priors from the masked ProtLMs (ESM-2) to guide combinatorial sequence space navigation. By prioritizing beneficial mutations through these learned priors, MCTS-HMSO achieves deeper exploration of the search tree with the same computational budget (i.e., iteration count), thereby enhancing exploration toward high-fitness regions.

Besides, as shown in **Table 1**, MCTS-HMSO also demonstrated consistent improvements in structural stability (pLDDT) and sequence plausibility (perplexity) compared to conventional MCTS. However, the magnitude of improvement was less pronounced than that observed in GA-HMSO. This discrepancy may arise from the inherent constraint of MCTS, which introduces only one mutation per iteration. Such a restriction limits the parallel multi-site exploration of combinatorial mutational spaces, thereby reducing convergence efficiency relative to genetic algorithm-based approaches that permit multi-site modifications in a single iteration.

## Discussion

ProtHMSO establishes a highly efficient mechanism for traversing the fitness landscape by leveraging the rich evolutionary priors within masked protein language models (ProtLMs), fully validating the rationality and effectiveness of employing implicit sequence constraints to guide the search strategy. Building upon this solid foundation, integrating explicit structural validation represents a significant avenue for further optimization. While ProtLMs implicitly capture structural contacts, inferences based on sequence statistics are not strictly equivalent to rigorous thermodynamic stability assessments; thus, a subtle distinction may remain between evolutionary plausibility and absolute physical stability. Integrating physics-based energy functions, such as Rosetta energy terms or molecular dynamics simulations, could significantly enhance prediction accuracy by serving as a rigorous filter to ensure structural integrity and stability. However, a critical challenge in this integration lies in the trade-off between computational efficiency and search depth; the high computational overhead of atomic-level physics calculations conflicts with the core advantage of ProtHMSO in rapidly traversing vast combinatorial spaces. To address this, a hybrid *coarse-to-fine* strategy represents a viable path for future optimization, where ProtHMSO acts as a high-throughput screener to narrow down the search space to high-potential candidates, while physics-based scoring functions are subsequently applied for fine-grained validation.

Beyond theoretical benchmarks, ProtHMSO exhibits substantial transformation potential in practical protein engineering scenarios, such as the rapid development of therapeutic peptides, industrial enzymes, and antibodies. Traditionally, the protein design process is heavily bottlenecked by the combinatorial explosion of multi-site mutations and the high costs of wet-lab screening. ProtHMSO significantly accelerates this pipeline through two perspectives. Computationally, it utilizes prior knowledge for heuristic search, significantly reducing the combinatorial search space and enabling the selection of documents to be reduced from millions to only tens or even hundreds of variations. Experimentally, it improves the conversion success rate in wet laboratories by enhancing the antimicrobial activity and antimicrobial capacity of antimicrobial peptides. Consequently, as a plug-and-play module that can be integrated into existing automated biofoundries or directed evolution workflows, ProtHMSO substantially increases the experimental hit rate and reduces the prohibitive costs associated with large-scale DNA synthesis and high-throughput screening, thereby accelerating the overall transition from computational design to real-world functional protein discovery.

## Conclusion

In this study, we proposed ProtHMSO, a heuristic multi-site optimization framework for protein sequence design guided by masked protein language models. Its core innovation lies in constructing a heuristic multi-site optimization framework based on a masked protein language model. Relying on the model’s dynamic perception of sequence context, it accurately captures and utilizes epistasis interactions between residues to guide heuristic search for co-mutations and iterative sequence optimization. This overcomes the core bottleneck of existing methods, which struggle to fully utilize nonlinear interaction information between residues to achieve efficient multi-site combinatorial mutation design. Furthermore, ProtHMSO can be further integrated into genetic algorithms and Monte Carlo tree search algorithms (GA-HMSO and MCTS-HMSO) to enhance evolutionary optimization. Experiments on AMP and ProteinGym datasets demonstrate that ProtHMSO consistently improves AMP functionality and activity, effectively explores beneficial mutations in long protein sequences, and accelerates convergence in heuristic frameworks. By coupling evolutionary search with language model–informed mutation guidance, ProtHMSO offers a robust and generalizable strategy for protein engineering while mitigating destabilizing and deleterious substitutions. Future work will explore scalability to larger proteins and integration with physics-based energy functions.

## Supporting information

S1 File Hyperparameter optimization and analysis. This supporting information file contains top-*k* selection, efficiency comparison of different methods, mutation probability parameters in GA-HMSO, group scoring strategies in MCTS-HMSO, analysis of the rationality of ProtHMSO mutations, robustness analysis of the experiment, and structural stability analysis of the mutants.
(PDF)

## References

[1] Dou Z, Sun Y, Jiang X, Wu X, Li Y, Gong B. Data-driven strategies for the computational design of enzyme thermal stability: trends, perspectives, and prospects: data-driven strategies for enzyme thermostability design. Acta Biochimica et Biophysica Sinica. 2023;55(3):343. 37143326 10.3724/abbs.2023033 PMC10160227

[2] Chu H, Tian Z, Hu L, Zhang H, Chang H, Bai J, et al. High-Temperature Tolerance Protein Engineering through Deep Evolution. Biodes Res. 2024;6:0031. doi: 10.34133/bdr.0031 38572349 PMC10988389

[3] Hie BL, Yang KK, Kim PS. Evolutionary velocity with protein language models predicts evolutionary dynamics of diverse proteins. Cell Syst. 2022;13(4):274-285.e6. doi: 10.1016/j.cels.2022.01.003 35120643

[4] Madani A, Krause B, Greene ER, Subramanian S, Mohr BP, Holton JM, et al. Large language models generate functional protein sequences across diverse families. Nat Biotechnol. 2023;41(8):1099–106. doi: 10.1038/s41587-022-01618-2 36702895 PMC10400306

[5] Yu H, Ma S, Li Y, Dalby PA. Hot spots-making directed evolution easier. Biotechnol Adv. 2022;56:107926. doi: 10.1016/j.biotechadv.2022.107926 35151790

[6] Zhang J, Zeng H, Chen J, Zhu Z. INAB: identify nucleic acid binding domain via cross-modal protein language models and multiscale computation. Brief Bioinform. 2025;26(5):bbaf509. doi: 10.1093/bib/bbaf509 41020638 PMC12477684

[7] Yan K, Lv H, Guo Y, Peng W, Liu B. sAMPpred-GAT: prediction of antimicrobial peptide by graph attention network and predicted peptide structure. Bioinformatics. 2023;39(1):btac715. doi: 10.1093/bioinformatics/btac715 36342186 PMC9805557

[8] Arslan M, Karadağ D, Kalyoncu S. Protein engineering approaches for antibody fragments: directed evolution and rational design approaches. Turk J Biol. 2019;43(1):1–12. doi: 10.3906/biy-1809-28 30930630 PMC6426644

[9] Luo J, Zhao K, Chen J, Yang C, Qu F, Liu Y, et al. iMFP-LG: Identify Novel Multi-functional Peptides Using Protein Language Models and Graph-based Deep Learning. Genomics Proteomics Bioinformatics. 2025;22(6):qzae084. doi: 10.1093/gpbjnl/qzae084 39585308 PMC12011362

[10] Yan K, Lv H, Shao J, Chen S, Liu B. TPpred-SC: multi-functional therapeutic peptide prediction based on multi-label supervised contrastive learning. Sci China Inf Sci. 2024;67(11). doi: 10.1007/s11432-024-4147-8

[11] Ding X, Zou Z, Brooks Iii CL. Deciphering protein evolution and fitness landscapes with latent space models. Nat Commun. 2019;10(1):5644. doi: 10.1038/s41467-019-13633-0 31822668 PMC6904478

[12] Yan K, Chen S, Liu B, Wu H. Accurate prediction of toxicity peptide and its function using multi-view tensor learning and latent semantic learning framework. Bioinformatics. 2025;41(9):btaf489. doi: 10.1093/bioinformatics/btaf489 40905623 PMC12457739

[13] Holland JH. Genetic Algorithms. Sci Am. 1992;267(1):66–72. doi: 10.1038/scientificamerican0792-661411454

[14] Singh J, Ator MA, Jaeger EP, Allen MP, Whipple DA, Soloweij JE, et al. Application of Genetic Algorithms to Combinatorial Synthesis: A Computational Approach to Lead Identification and Lead Optimization,. J Am Chem Soc. 1996;118(7):1669–76. doi: 10.1021/ja953172i

[15] Yan K, Yu H, Chen S, Shaytan AK, Liu B, Wang Y. DSCA-HLAII: A dual-stream cross-attention model for predicting peptide-HLA class II interaction and presentation. PLoS Comput Biol. 2026;22(1):e1013836. doi: 10.1371/journal.pcbi.1013836 41481588 PMC12758783

[16] Yokobayashi Y, Ikebukuro K, McNiven S, Karube I. Directed evolution of trypsin inhibiting peptides using a genetic algorithm. J Chem Soc, Perkin Trans 1. 1996;(20):2435. doi: 10.1039/p19960002435

[17] Qu F, Zhao Y, Huang T, Wang X, Zhang J, Chen J. HiPHD: Hierarchical Classification for Protein Remote Homology Detection by Incorporating Protein Sequential and Structural Information. IEEE Trans Comput Biol Bioinform. 2025;22(6):2872–81. doi: 10.1109/TCBBIO.2025.3604441 40889305

[18] Yoshida M, Hinkley T, Tsuda S, Abul-Haija YM, McBurney RT, Kulikov V, et al. Using evolutionary algorithms and machine learning to explore sequence space for the discovery of antimicrobial peptides. Chem. 2018;4(3):533–43.

[19] Zhang H, Wang Y, Zhu Y, Huang P, Gao Q, Li X, et al. Machine learning and genetic algorithm-guided directed evolution for the development of antimicrobial peptides. J Adv Res. 2025;68:415–28. doi: 10.1016/j.jare.2024.02.016 38431124 PMC11785909

[20] Wang Y, Tang H, Huang L, Pan L, Yang L, Yang H, et al. Self-play reinforcement learning guides protein engineering. Nat Mach Intell. 2023;5(8):845–60. doi: 10.1038/s42256-023-00691-9

[21] Liu Y, Zhu Y, Wang J, Hu R, Shen C, Qu W, et al. A Multi-Objective Molecular Generation Method Based on Pareto Algorithm and Monte Carlo Tree Search. Adv Sci (Weinh). 2025;12(20):e2410640. doi: 10.1002/advs.202410640 40183315 PMC12120794

[22] Wang Y, Yu L, Shao J, Zhu Z, Zhang L. Structure-driven protein engineering for production of valuable natural products. Trends Plant Sci. 2023;28(4):460–70. doi: 10.1016/j.tplants.2022.11.004 36473772

[23] Liao X, Wang Q, Liang Z, Xiao L, Chen J. DualMPNN: Harnessing Structural Alignments for High-Recovery Inverse Protein Folding. The Thirty-Ninth Annual Conference on Neural Information Processing Systems;.

[24] Wang L, Qiu C, Xiao L, Chen J. PepPFDPO: Multi-Objective Antimicrobial Peptide Generation using Pareto-Frontier Enhanced Direct Preference Optimization. In: 2025 IEEE International Conference on Bioinformatics and Biomedicine (BIBM), 2025. 01–9. 10.1109/bibm66473.2025.11356223

[25] Xu X, Xu T, Zhou J, Liao X, Zhang R, Wang Y, et al. AB-Gen: Antibody Library Design with Generative Pre-trained Transformer and Deep Reinforcement Learning. Genomics Proteomics Bioinformatics. 2023;21(5):1043–53. doi: 10.1016/j.gpb.2023.03.004 37364719 PMC10928431

[26] Lan T, Su S, Ping P, Hutvagner G, Liu T, Pan Y, et al. Generating mutants of monotone affinity towards stronger protein complexes through adversarial learning. Nat Mach Intell. 2024;6(3):315–25. doi: 10.1038/s42256-024-00803-z

[27] Cai H, Du B, Luo Y, Ouyang S, Su K, Zhang L. MMSite: A Multi-modal Framework for the Identification of Active Sites in Proteins. In: Advances in Neural Information Processing Systems 37, 2024. 45819–49. 10.52202/079017-1457

[28] Cheng P, Mao C, Tang J, Yang S, Cheng Y, Wang W, et al. Zero-shot prediction of mutation effects with multimodal deep representation learning guides protein engineering. Cell Res. 2024;34(9):630–47. doi: 10.1038/s41422-024-00989-2 38969803 PMC11369238

[29] Vaswani A, Shazeer N, Parmar N, Uszkoreit J, Jones L, Gomez AN. Attention is all you need. Advances in neural information processing systems. 2017;30.

[30] Suzek BE, Wang Y, Huang H, McGarvey PB, Wu CH, UniProt Consortium. UniRef clusters: a comprehensive and scalable alternative for improving sequence similarity searches. Bioinformatics. 2015;31(6):926–32. doi: 10.1093/bioinformatics/btu739 25398609 PMC4375400

[31] Lin Z, Akin H, Rao R, Hie B, Zhu Z, Lu W, et al. Evolutionary-scale prediction of atomic-level protein structure with a language model. Science. 2023;379(6637):1123–30. doi: 10.1126/science.ade2574 36927031

[32] Elnaggar A, Heinzinger M, Dallago C, Rehawi G, Wang Y, Jones L, et al. ProtTrans: Toward Understanding the Language of Life Through Self-Supervised Learning. IEEE Trans Pattern Anal Mach Intell. 2022;44(10):7112–27. doi: 10.1109/TPAMI.2021.3095381 34232869

[33] Watson JL, Juergens D, Bennett NR, Trippe BL, Yim J, Eisenach HE, et al. De novo design of protein structure and function with RFdiffusion. Nature. 2023;620(7976):1089–100. doi: 10.1038/s41586-023-06415-8 37433327 PMC10468394

[34] Dauparas J, Anishchenko I, Bennett N, Bai H, Ragotte RJ, Milles LF, et al. Robust deep learning-based protein sequence design using ProteinMPNN. Science. 2022;378(6615):49–56. doi: 10.1126/science.add2187 36108050 PMC9997061

[35] Alamdari S, Thakkar N, van den Berg R, Lu A, Fusi N, Amini A. Protein generation with evolutionary diffusion: sequence is all you need. In:

[36] Wang Y, Zhang Q, Qin M, Zhuang X, Li X, Gong Z. Knowledge-aware reinforced language models for protein directed evolution. In: 2024.

[37] Devlin J, Chang MW, Lee K, Toutanova K. Bert: Pre-training of deep bidirectional transformers for language understanding. arXiv preprint. 2018. https://arxiv.org/abs/1810.04805

[38] Jumper J, Evans R, Pritzel A, Green T, Figurnov M, Ronneberger O, et al. Highly accurate protein structure prediction with AlphaFold. Nature. 2021;596(7873):583–9. doi: 10.1038/s41586-021-03819-2 34265844 PMC8371605

[39] Tran TVT, Hy TS. Protein Design by Directed Evolution Guided by Large Language Models. IEEE Trans Evol Computat. 2025;29(2):418–28. doi: 10.1109/tevc.2024.3439690

[40] Li J, Wu Z, Lin W, Luo J, Zhang J, Chen Q, et al. iEnhancer-ELM: improve enhancer identification by extracting position-related multiscale contextual information based on enhancer language models. Bioinform Adv. 2023;3(1):vbad043. doi: 10.1093/bioadv/vbad043 37113248 PMC10125906

[41] Small BG, McColl BW, Allmendinger R, Pahle J, López-Castejón G, Rothwell NJ, et al. Efficient discovery of anti-inflammatory small-molecule combinations using evolutionary computing. Nat Chem Biol. 2011;7(12):902–8. doi: 10.1038/nchembio.689 22020553 PMC3223407

[42] Browne CB, Powley E, Whitehouse D, Lucas SM, Cowling PI, Rohlfshagen P, et al. A Survey of Monte Carlo Tree Search Methods. IEEE Trans Comput Intell AI Games. 2012;4(1):1–43. doi: 10.1109/tciaig.2012.2186810

[43] Cappé O, Garivier A, Maillard O-A, Munos R, Stoltz G. Kullback–Leibler upper confidence bounds for optimal sequential allocation. Ann Statist. 2013;41(3). doi: 10.1214/13-aos1119

[44] Kikkawa N, Ohno H. Unified theory of upper confidence bound policies for bandit problems targeting total reward, maximal reward, and more. In: 2024. https://doi.org/arXiv:241100339

[45] Zhang H, Yu T. AlphaZero. Deep Reinforcement Learning: Fundamentals, Research and Applications. 2020; p. 391–415.

[46] Meier J, Rao R, Verkuil R, Liu J, Sercu T, Rives A. Language models enable zero-shot prediction of the effects of mutations on protein function. Advances in Neural Information Processing Systems. 2021;34:29287–303.

[47] Fang Y, Chen J, Wang H, Wang S, Chang M, Chen Q, et al. Integrating large-scale single-cell RNA sequencing in central nervous system disease using self-supervised contrastive learning. Commun Biol. 2024;7(1):1107. doi: 10.1038/s42003-024-06813-2 39251817 PMC11383967

[48] Pirtskhalava M, Amstrong AA, Grigolava M, Chubinidze M, Alimbarashvili E, Vishnepolsky B, et al. DBAASP v3: database of antimicrobial/cytotoxic activity and structure of peptides as a resource for development of new therapeutics. Nucleic Acids Res. 2021;49(D1):D288–97. doi: 10.1093/nar/gkaa991 33151284 PMC7778994

[49] Capecchi A, Cai X, Personne H, Köhler T, van Delden C, Reymond J-L. Machine learning designs non-hemolytic antimicrobial peptides. Chem Sci. 2021;12(26):9221–32. doi: 10.1039/d1sc01713f 34349895 PMC8285431

[50] Liu X, Luo J, Wang X, Zhang Y, Chen J. Directed evolution of antimicrobial peptides using multi-objective zeroth-order optimization. Brief Bioinform. 2024;26(1):bbae715. doi: 10.1093/bib/bbae715 39800873 PMC11725395

[51] Dias M, Franceschi D, Frazer J, Gal Y, Kollasch A, Marks D, et al. ProteinGym: Large-Scale Benchmarks for Protein Fitness Prediction and Design. In: Advances in Neural Information Processing Systems 36, 2023. 64331–79. 10.52202/075280-2810

[52] Kingma DP, Mohamed S, Jimenez Rezende D, Welling M. Semi-supervised learning with deep generative models. Advances in Neural Information Processing Systems. 2014;27.

[53] Nijkamp E, Ruffolo JA, Weinstein EN, Naik N, Madani A. ProGen2: Exploring the boundaries of protein language models. Cell Syst. 2023;14(11):968-978.e3. doi: 10.1016/j.cels.2023.10.002 37909046

[54] Wang J, Chitsaz F, Derbyshire MK, Gonzales NR, Gwadz M, Lu S, et al. The conserved domain database in 2023. Nucleic Acids Res. 2023;51(D1):D384–8. doi: 10.1093/nar/gkac1096 36477806 PMC9825596

[55] Marchler-Bauer A, Bo Y, Han L, He J, Lanczycki CJ, Lu S, et al. CDD/SPARCLE: functional classification of proteins via subfamily domain architectures. Nucleic Acids Res. 2017;45(D1):D200–3. doi: 10.1093/nar/gkw1129 27899674 PMC5210587

