# Supplementary material for: Heuristic multi-site optimization for protein sequence design using Masked Protein Language Models
Source: PLoS Comput Biol. 2026 Jun 5;22(6):e1014365. doi: 10.1371/journal.pcbi.1014365 (PMC13252849; doi:10.1371/journal.pcbi.1014365)
Supplement: S1 File — This supporting information file contains top-k selection, efficiency comparison of different methods, mutation probability parameters in GA-HMSO, group scoring strategies in MCTS-HMSO, analysis of the rationality of ProtHMSO mutations, robustness analysis of the experiment, and structural stability analysis of the mutants. (PDF) [file pcbi.1014365.s001.pdf]

Supplementary Information: Heuristic Multi-site Optimization for Protein Sequence Design using Masked Protein Language Models

Lijuan Wang[1], Yuze Wang[2], Chen Qiu[1], Liwei Xiao[1], Xianliang Liu[1], Junjie Chen [1,*]

[1] School of Computer Science and Technology, Harbin Institute of Technology, Shenzhen, Guangdong, China;
[2] School of Computer Science and Technology, Harbin Institute of Technology, Weihai, Shandong, China
* Corresponding author: Junjie Chen; Email: junjiechen@hit.edu.cn.

S.1 Top-k Selection

To systematically evaluate the impact of Top-k selection on model performance during the ESM2 mutation process, we utilized the *case3* dataset, which exhibited the poorest initial AMP performance, as a benchmark for conducting five-site directed mutation experiments. We compared the outcomes where k was set to 1, 3, and 5. The probability value distributions illustrated in Figure S1 reveal distinct trends in performance across different k values. Specifically, the results at $k = 1$ (Mean: 0.87 for AMP) did not show a substantial deviation from those observed at $k = 3$ (Mean: 0.91 for AMP) or $k = 5$ (Mean: 0.92 for AMP). Furthermore, a comparison between $k = 3$ and $k = 5$ indicates that while increasing k theoretically expands the candidate pool, the difference in probability distributions is negligible, suggesting that performance gains have reached a plateau. Given the nature of heuristic search algorithms, balancing computational efficiency with search space size is critical; as k increases, the search space expands significantly without yielding proportional improvements in performance. Consequently, to optimize the trade-off between prediction accuracy and computational cost, selecting a k value between 1 and 3 is recommended for ESM2-based mutation predictions. This range ensures robust predictive efficacy while effectively managing the complexity of the search space.

S.2 Efficiency Comparison of Different Methods

To comprehensively evaluate the computational efficiency of different mutation strategies, we conducted a detailed runtime analysis using the challenging *case3* dataset. When using ProtHMSO for mutation prediction on NVIDIA A100 GPUs, we set the batch size to 32. The larger the batch size, the faster the mutation prediction speed.

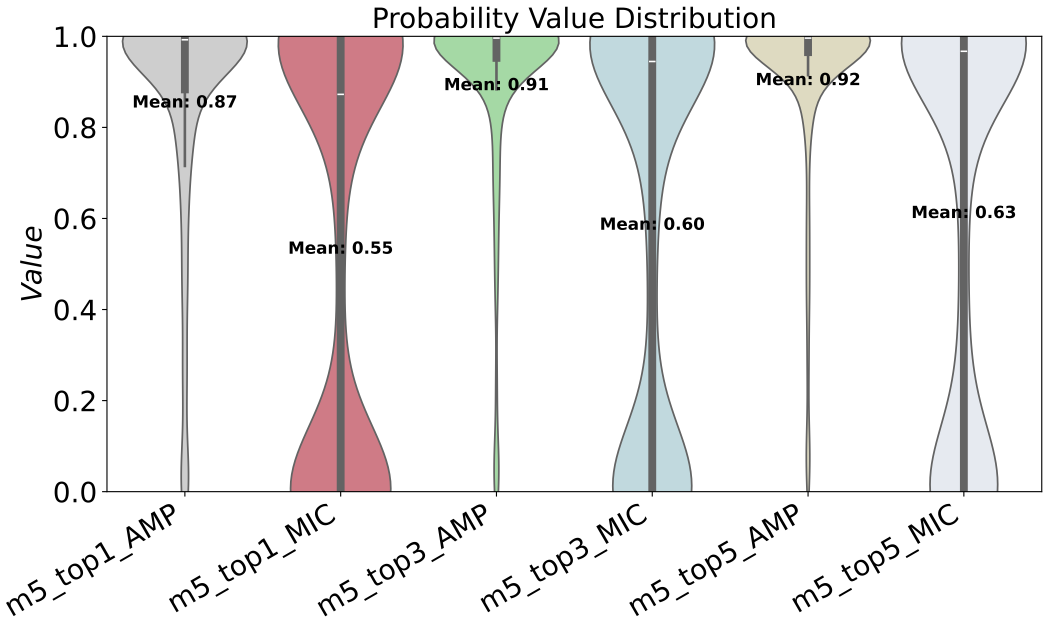

Figure S1: **Distribution of AMP and MIC probabilities for 5-site mutation variants (m5).** The variants were generated by sampling amino acids from the top k highest substitution probabilities at the target sites (denoted as top-k, where $k \in \{1, 3, 5\}$). White markers indicate the mean values for each distribution.

When performing genetic algorithm mutation, traditional algorithms mutate the sequence sequentially, while GA-HMSO processes mutations in parallel with a batch size of 32. The MCTS algorithm cannot use parallel mutation due to its inherent limitations.

We first compared the performance of Random mutation, the traditional GA, and the proposed ProtHMSO method when targeting 3 to 5 mutation sites. As presented in Table S1, the runtime for Random strategies (M3 to M5) ranged between 44 and 51 seconds, while the traditional GA required approximately 33.28 seconds. In contrast, our proposed ProtHMSO-based method (M3 to M5) demonstrated superior efficiency, with runtime consistently stabilizing around 20 seconds. This performance is significantly faster than both random mutation and the traditional GA, indicating that our approach effectively reduces computational costs while maintaining performance in fixed-site mutation tasks.

In the MCTS framework comparisons, the MCTS-HMSO$_{100}$ variant required 719.86 seconds compared to 417.79 seconds for the baseline. However, this increased computational cost is a worthwhile trade-off for the substantial gains in performance, as MCTS-HMSO successfully identifies high-fitness sequences that the baseline fails to discover. The observed latency is primarily dictated by the intrinsic sequential nature of the MCTS algorithm itself. Unlike population-based methods that allow for parallel processing, MCTS expands the search tree node-by-node, necessitating serial inference calls that preclude the batched acceleration typically used for ProtLMs. Therefore, the runtime difference reflects the structural constraints of the MCTS search strategy rather than an inefficiency in the HMSO method, confirming that the superior generation quality is achieved through a rigorous, albeit computationally demanding, step-by-step guidance.

Table S1: **Comparison of runtime efficiency for different mutation and search strategies on the case3 dataset.** The columns labeled *M3*, *M4*, and *M5* represent experiments where 3, 4, and 5 amino acid sites were mutated, respectively. *MCTS_100* denotes the Monte Carlo Tree Search algorithm run with a budget of 100 iterations. The runtime is measured in seconds (s).

**Method**	**Random**			**ProtHMSO**			**GA**	**GA-HMSO**	**MCTS_100**	**MCTS-HMSO_100**
	**M3**	**M4**	**M5**	**M3**	**M4**	**M5**				
**Time (s)**	44.96	51.28	48.08	20.90	20.20	20.33	33.28	24.48	417.79	719.86

S.3 Mutation Probability Parameters in GA-HMSO

To determine the optimal configuration for mutation probability parameters within GA, we systematically evaluated the impact of iteration thresholds and various mutation probability strategies on the predicted probability distribution of generated sequences. First, regarding the selection of the iteration threshold, we compared its performance with thresholds set at 5, 7, 10, and 15 (as shown in Figure S2). The results indicate that an iteration threshold of 5 yields the highest mean probability for AMPs (Mean: 0.90), while maintaining a favorable balance in the probability distribution related to MIC. As the threshold increases to 10 or 15, although some samples retain high prediction scores, the overall mean of the distribution shows a downward trend, accompanied by an increase in dispersion. Consequently, we fixed the iteration threshold at 5 for subsequent experiments to ensure the algorithm could rapidly converge to a high-quality solution space in the early stages.

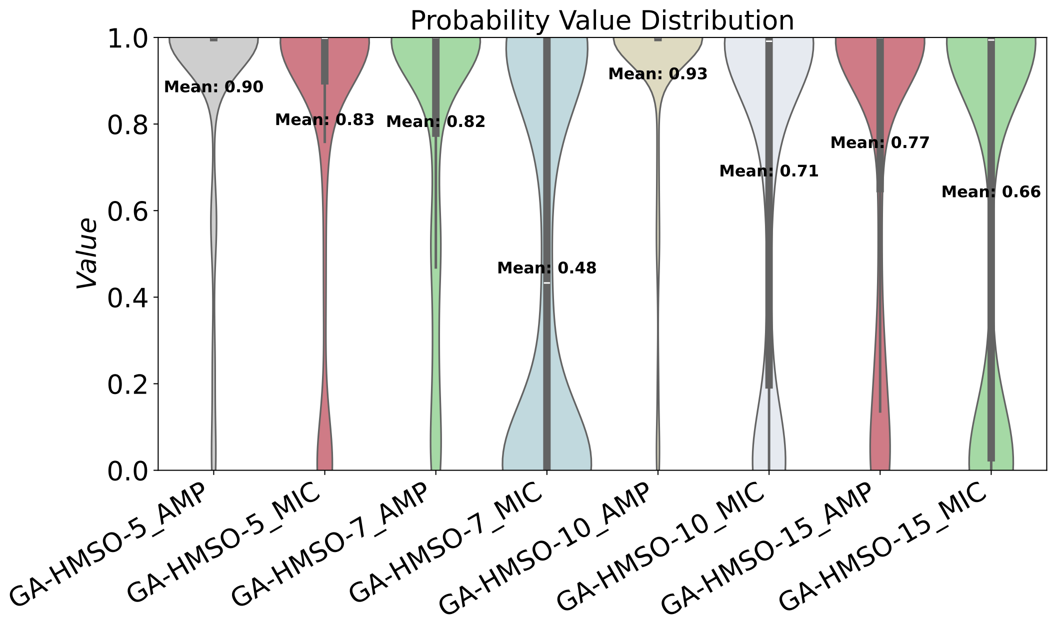

Figure S2: **Probability value distributions for different iteration thresholds.** The x-axis labels indicate the specific iteration threshold (5, 7, 10, or 15) applied in the GA-HMSO algorithm for AMP and MIC predictions.

Building on this, we further investigated the influence of mutation probability strate-

gies on algorithmic performance by comparing three modes: fixed low probability, fixed high probability, and dynamic probability. Results in Figure S3 reveal that the fixed low probability strategy (GA-HMSO-low) limits the diversity of generated sequences, resulting in a lower overall mean probability (AMP Mean: 0.29). Conversely, while the fixed high probability strategy enhances exploration, it risks disrupting established superior structures. In contrast, the dynamic probability strategy (GA-HMSO-dynamic) demonstrates the best comprehensive performance, achieving an AMP probability mean of 0.90 while maintaining a high MIC-related probability (Mean: 0.83). This dynamic adjustment mechanism effectively balances the algorithm's global exploration and local exploitation capabilities, thereby generating sequences with higher predicted activity and superior property distributions.

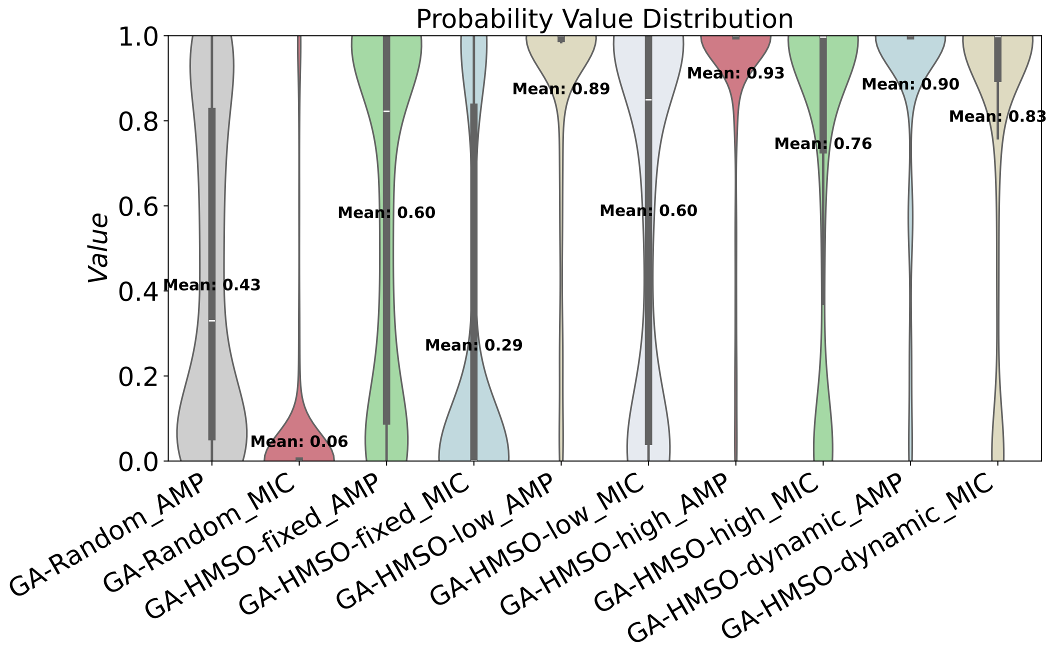

Figure S3: **Comparison of probability distributions across varying mutation settings.** The x-axis labels denote the specific mutation strategy applied: **fixed** uses a constant probability, **dynamic** employs an adaptive probability, while **high** and **low** represent mutation probabilities maintained at consistently high or low levels throughout the process.

S.4 Group Scoring Strategies in MCTS-HMSO

We evaluated two distinct group scoring strategies to determine the optimal method for assessing the performance of nodes within a group: selecting the maximum UCB value of visited nodes within the group (*max*), and calculating the weighted average of visited nodes (*sum*). To verify the efficacy of these strategies, we conducted experiments across varying numbers of iterations (100, 300, and 500) and analyzed the resulting probability distributions for AMP and MIC (as shown in the figure S4).

The experimental results demonstrate that the performance of these two strategies is highly comparable across different iteration stages. Regardless of whether the iteration

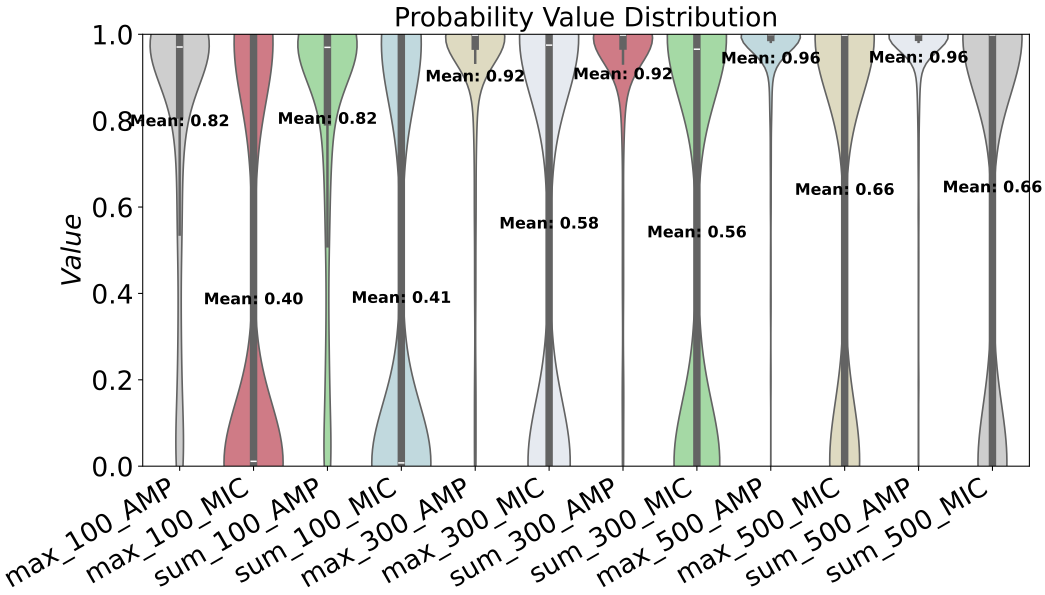

Figure S4: **Comparison of group scoring mechanisms and iteration counts in the MCTS-HMSO algorithm.** ***max*** represents the group score using the maximum value of explored nodes within the group, while ***sum*** utilizes the weighted sum of the explored nodes. The numerical values (100, 300, 500) denote the number of iterations performed.

count was 100, 300, or 500, there were no significant differences in the shape or the mean values of the probability distributions generated by the *max* and *sum* strategies. For instance, at 500 iterations, the *max* strategy yielded a mean AMP probability of 0.96 and a mean MIC probability of 0.66, which were identical to those obtained using the *sum* strategy. This suggests that MCTS-HMSO possesses a degree of robustness regarding the specific aggregation method used for group scoring. However, considering that the weighted average provides a more comprehensive reflection of the overall quality of all explored nodes within a group, rather than relying solely on a single optimal node, thereby potentially offering more stable guidance. We ultimately selected the weighted average as the standard method for group scoring in this study.

S.5 Analysis of the rationality of ProtHMSO mutation

To investigate the rationality of mutation site selection by the ESM2 model, we analyzed the highest (Top) and lowest (Bottom) probability mutants of the sequence *KVPIGAIKKGGKIIKKGLGVIGAAGTAHEVYSHVKNRH* (Figure S5).

The Top sequence demonstrates a strong tendency to optimize AMP characteristics. It eliminates helix-breaking proline residues to enhance conformational stability and incorporates lysine mutations (e.g., N34K) to increase the net positive charge, thereby facilitating electrostatic interactions with negatively charged bacterial membranes. Furthermore, the sequence retains core hydrophobic segments, preserving the amphipathic α-helical architecture essential for membrane insertion and pore formation.

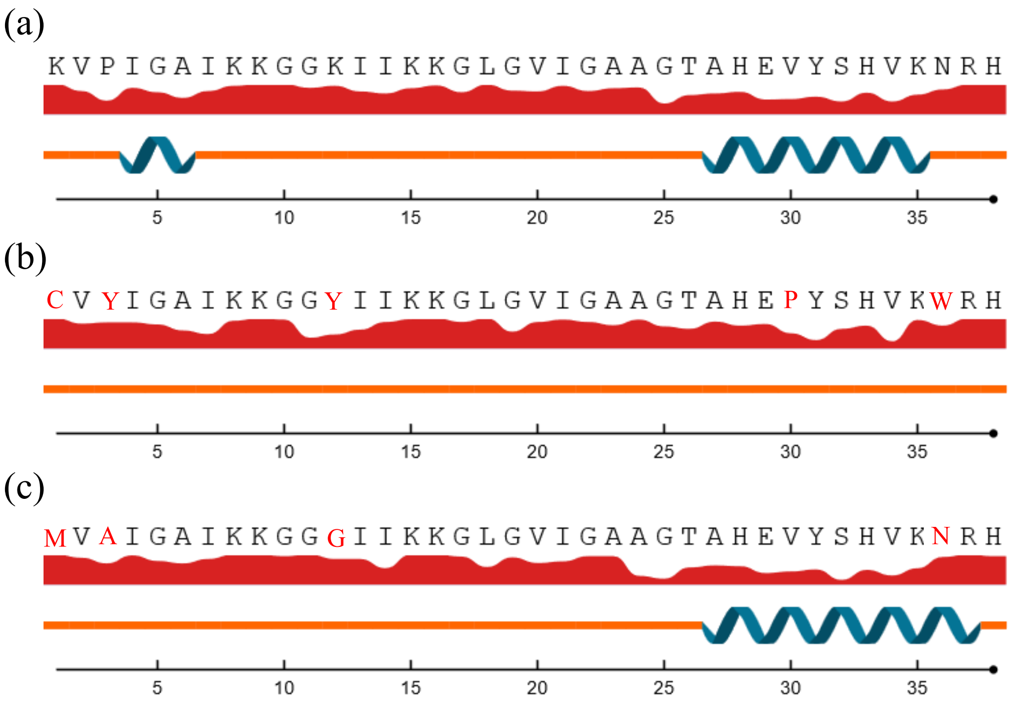

Figure S5: Comparative analysis of secondary structures for the original sequence and ProtHMSO-predicted mutants. The red amino acids represent mutant amino acids. (a) Two-dimensional structure of the original sequence. (b) Two-dimensional structure of the sequence with the lowest mutation probability. (c) Two-dimensional structure of the sequence with the highest mutation probability.

Conversely, the Bottom sequence reflects the model's sensitivity to deleterious mutations. It introduces cysteines, which pose a risk of non-specific disulfide aggregation, and accumulates bulky aromatic residues (Tyrosine, Tryptophan) that cause excessive hydrophobicity and steric hindrance. Consequently, the characteristic α-helical structure completely degenerates into a random coil, disrupting the amphipathic balance required for function. This stark contrast confirms that ESM2 has effectively internalized the complex mapping between sequence patterns, structural stability, and physicochemical properties.

S.6 Robustness assessment experiment

To verify the robustness of our evaluation method under noisy predictors, we specifically selected the most challenging and representative Case 3 dataset for supplementary experiments. In the specific experimental setup, we superimposed Gaussian noise with a mean of 0 and a standard deviation of 0.05 onto the prediction probability results of the original AMP and MIC classification models, and strictly limited the final scores to a reasonable range of [0, 1] to simulate a fitness model with noise. The experimental results (as shown in Figure S6) indicate that, with the introduction of noise interference (groups marked "_N" in the figure), the score distribution and mean of each model variant on the function enhancement and activity enhancement tasks showed a smaller

fluctuation compared to the original noise-free model (for example, the mean of the best-performing ProtHMSO_M5 model slightly decreased from 0.86 to 0.85 in the function enhancement task and from 0.56 to 0.55 in the activity enhancement task). This result demonstrates that our method does exhibit some fluctuation when facing fitness models of different qualities or with noise, but the degree of fluctuation is relatively small.

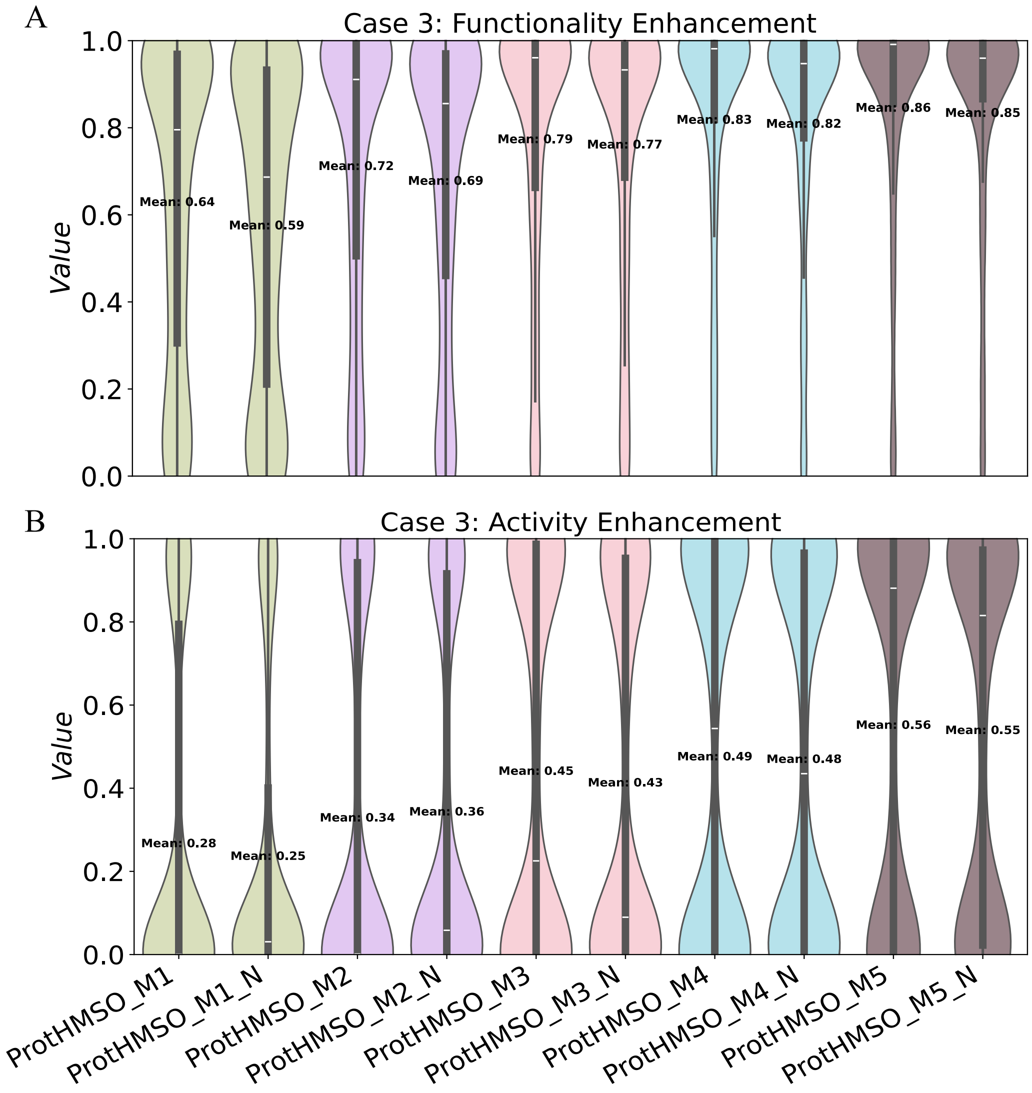

Figure S6: Comparison of AMP and MIC probability values of different methods. ProtHMSO indicates a variant generated using ESM-2, N indicates a variant generated after adding noise to the predictor, M1 indicates a mutation at one site, and so on.

S.7 Structural stability assessment

To further validate the biophysical properties of the generated variants, we supplemented the study with explicit structure and energy assessment experiments specifically for the most challenging and performance-representing Case 3 dataset (which requires simultaneous optimization of function and activity). In the experimental pro-

cedure, we first used AlphaFold to predict the 3D structures of the original sequence and variants generated by different mutation strategies, and then used PyRosetta to calculate the precise energy values of these predicted conformations. Generally, for short peptide molecules, an energy value between -10 and 5 is considered a usable conformation with reasonable biophysical properties and structural stability. As shown in Figure S7, the energy distribution of the variants generated by ProtHMSO mostly falls within the normal and reasonable range of [-10, 5]. Furthermore, as the number of mutation sites increases (from M1 to M5), the ProtHMSO-guided mutants exhibit a lower (i.e., more stable) average energy value than random mutations. For example, with 5 site mutations (M5), the average energy of the ProtHMSO variant decreased to 3.34, which is not only significantly better than random mutations under the same conditions (average 4.91), but also better than the original sequence in Case 3 (average 5.06). This result demonstrates that ProtHMSO, by virtue of the evolutionary priors implied in the language model, can effectively overcome the epistatic effects caused by multi-site mutations, and while significantly improving sequence fitness (function and activity), it can still maintain or even optimize the thermodynamic stability of its native conformation.

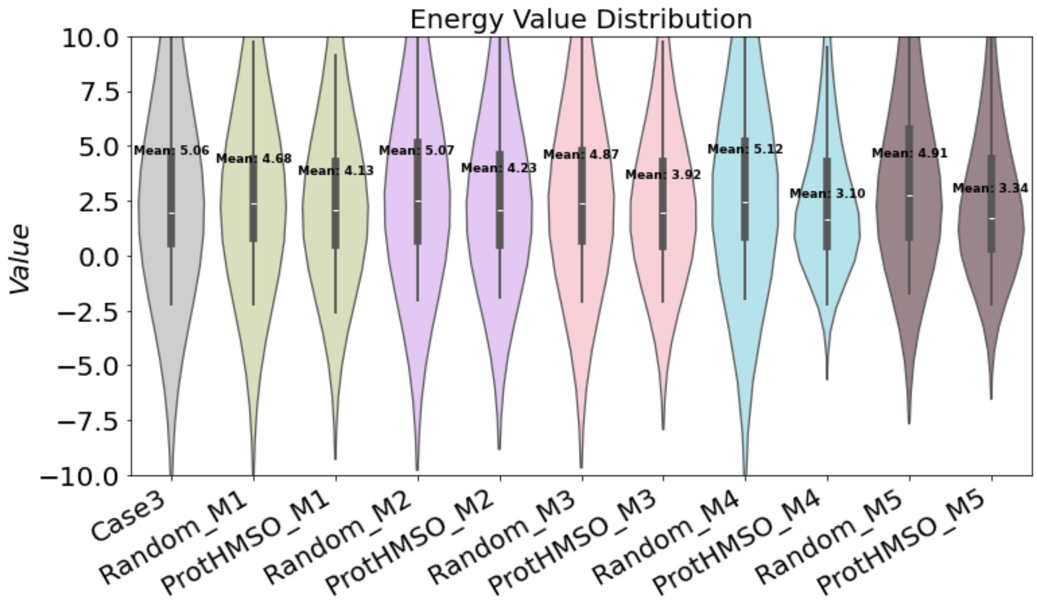

Figure S7: Comparison of AMP and MIC probability values of different methods. Case3 represents the energy value distribution of the original dataset, Random represents the energy value distribution of the variant generated using random mutations, ProtHMSO represents the energy value distribution of the variant generated using ESM-2, M1 represents a mutation at one site, and so on.
