# Supplementary material for: Heuristic multi-site optimization for protein sequence design using Masked Protein Language Models
Source: PLoS Comput Biol. 2026 Jun 5;22(6):e1014365. doi: 10.1371/journal.pcbi.1014365 (PMC13252849; doi:10.1371/journal.pcbi.1014365)
Supplement: Attachment — Submitted filename: Response Letter for ProtHMSO.docx [file pcbi.1014365.s003.docx]

Dear editor,

Thanks for your feedback and the attached review comments from three reviewers. Their constructive comments are very helpful for further strengthening the presentation of our paper. As suggested, the manuscript has been carefully revised. Our point-to-point responses can be summarized as follows. For clarity, the reviewers’ comments are colored in green beginning with **C** (comment), while our responses in blue beginning with **R** (response). All the revised texts in the manuscript are highlighted with red.

**To Reviewer #1**

**C-overall**: This paper proposed ProtHMSO, a heuristic multi-site optimization framework. ProtHMSO mimics natural evolutionary mechanism by employing protein language models (ProtLM)-derived substitution probabilities to heuristically search for rational mutations. ProtHMSO can also be integrated into GA and MCTS algorithms to improve their optimization performance. Benchmark experiments demonstrate that ProtHMSO exhibits a SOTA performance compared with baseline methods. This paper is novel, providing an efficient strategy for protein sequence optimization.

Strengths:

1. Introduce a heuristic multi-site optimization framework to guide the selection of multi-site mutations, breaking through the limitation of traditional single-point mutations that ignore epistatic interactions between sites.

2. Integrate into the genetic algorithm (GA) and Monte Carlo tree search (MCTS), enhancing their convergence efficiency.

3. Achieve a SOTA performance on multiple datasets, verifying its effectiveness in different lengths and scenarios.

**R-overall:** Thank you for your careful review with regard to our manuscript. We have made comprehensive changes according to your suggestions.

**C-1.** The challenge faced by existing multi-site optimization should be highlighted, and the motivation of this work can be further emphasized in the introduction.

**R-1.** We have revised paragraphs 3, 4, and 5 of the introduction section of the paper. The revised content was highlighted with red in the main manuscript.

**C-2.** It is better to analyze the impact of the k value in top-k selection in Algorithm 1.

**R-2.** To systematically evaluate the impact of Top-k selection on model performance during the ESM2 mutation process, we utilized the *case3* dataset, which exhibited the poorest initial antimicrobial peptide performance, as a benchmark for conducting five-site directed mutation experiments. We compared the outcomes where *k* was set to 1, 3, and 5, with the resulting probability value distributions illustrated in the Fig S1 in supplementary information S.1.

An analysis of the probability distribution reveals distinct trends in performance across different *k* values. Specifically, the results obtained when *k=1* (Mean: 0.87 for AMP) did not show a substantial deviation from those observed at *k=3* (Mean: 0.91 for AMP) or *k=5* (Mean: 0.92 for AMP). Furthermore, a comparison between *k=3* and *k=5* indicates that while increasing *k* theoretically expands the candidate pool, the difference in probability distributions is negligible, suggesting that performance gains have reached a plateau. Given the nature of heuristic search algorithms, balancing computational efficiency with search space size is critical; as *k* increasing, the search space expands significantly without yielding proportional improvements in performance. Consequently, to optimize the trade-off between prediction accuracy and computational cost, selecting a *k* value between 1 and 3 is recommended for ESM2-based mutation predictions. This range ensures robust predictive efficacy while effectively managing the complexity of the search space.

**Fig S1. Distribution of AMP and MIC probabilities for 5-site mutation variants (m5).** The variants were generated by sampling amino acids from the top *k* highest substitution probabilities at the target sites (denoted as top-k, where $k\in\{1,3,5\}$).

**To Reviewer #2**

**C-overall**: This paper proposed a heuristic multi-site optimization framework, ProtHMSO, for protein sequence design. Its key innovation lies in leveraging evolutionary priors from masked language models (ESM-2) to guide the search process, effectively overcoming the limitations of conventional blind search methods. ProtHMSO can be used as standalone for directed optimization and as a plugin to enhance GAs and MCTS. Experiments on AMP dataset and ProteinGym dataset show that ProtHMSO achieves faster convergence and better results on benchmark datasets, and it is especially effective in challenging cases where both activity and functionality needed improvement.The manuscript is novel and well-written.

**R-overall:** Thank you for your critical assessment of our work. We have carefully addressed each of your concerns. The manuscript has been significantly improved with extensive revisions, supplemented by new experiments and expanded discussions.

**C-1.** The protein language models, genetic algorithm and Monte Carlo tree search have been widely used in various fields. Therefore, their introduction in related work section could be more concise.

**R-1.** We have streamlined the content of the Preliminary Knowledge and Related Work section. Specifically, for the protein language model section, we have removed the formula descriptions and focused on an overview of the latest models and the models used in this paper. For the genetic algorithm section, we have removed the detailed overview of traditional algorithms and focused on explaining the advantages and disadvantages of genetic algorithms and their optimization in this paper. For the MCTS algorithm, we have removed the traditional operation descriptions and focused on introducing the application of MCTS in sequences.

**C-2.** The AMP data was split into four disjoint subsets, but the reason how to choose the thresholds is unclear.

**R-2.** To comprehensively evaluate the model's capacity to improve peptide performance under varying initial conditions, we adopted the dataset partitioning strategy described by *Liu et al*. [1] Furthermore, to facilitate the generation of peptides with enhanced activity, we increased the probability threshold $P_{MIC}$ to 0.8. We added a note to the first paragraph of the Antimicrobial Peptide Dataset section of the paper.

Reference

[1] Liu X, Luo J, Wang X, Zhang Y, Chen J. Directed evolution of antimicrobial peptides using multi-objective zeroth-order optimization. Briefings in Bioinformatics. 2024;26(1).

**C-3.** While ProtHMSO achieves faster convergence, running inference on a large model like ESM-2 (650M) for every mutation step is computationally intensive. The hardware and total cost time should be given to help the users evaluate the practical trade-offs of using a large model in specific scenarios.

**R-3.** To comprehensively evaluate the computational efficiency of different mutation strategies, we conducted a detailed runtime analysis using the challenging *case3* dataset. When using ProtHMSO for mutation prediction on NVIDIA A100 GPUs, we set the batch size to 32. The larger the batch size, the faster the mutation prediction speed. When performing genetic algorithm mutation, traditional algorithms mutate the sequence sequentially, while GA-HMSO processes mutations in parallel with a batch size of 32. The MCTS algorithm cannot use parallel mutation due to its inherent limitations.

We first compared the performance of Random strategy, the traditional GA, and the proposed ProtHMSO method when targeting 3 to 5 mutation sites. As presented in Table S1 in Supplementary Information, the runtime for Random strategies (M3 to M5) ranged between 44 and 51 seconds, while the traditional GA required approximately 33.28 seconds. In contrast, our proposed ProtHMSO-based method (M3 to M5) demonstrated superior efficiency, with runtime consistently stabilizing around 20 seconds. This performance is significantly faster than both random mutation and the traditional GA, indicating that our approach effectively reduces computational costs while maintaining performance in fixed-site mutation tasks.

In the MCTS framework comparisons, the MCTS-HMSO_100 variant required 719.86 seconds compared to 417.79 seconds for the baseline. However, this increased computational cost is a worthwhile trade-off for the substantial gains in performance, as MCTS-HMSO successfully identifies high-fitness sequences that the baseline fails to discover. The observed latency is primarily dictated by the intrinsic sequential nature of the MCTS algorithm itself. Unlike population-based methods that allow for parallel processing, MCTS expands the search tree node-by-node, necessitating serial inference calls that preclude the batched acceleration typically used for ProtLMs. Therefore, the runtime difference reflects the structural constraints of the MCTS search strategy rather than an inefficiency in the HMSO method, confirming that the superior generation quality is achieved through a rigorous, albeit computationally demanding, step-by-step guidance.

**Table S1.** **Comparison of runtime efficiency for different mutation and search strategies on the case3 dataset.** The columns labeled *M3*, *M4*, and *M5* represent experiments, where 3, 4, and 5 amino acid sites were mutated, respectively. *MCTS_100* denotes the Monte Carlo Tree Search algorithm run with a budget of 100 iterations. The runtime is measured in seconds (s).

| Method | Random | | | | | | | | ProtHMSO | | | | | |  |
| --- | --- | --- | --- | --- | --- | --- | --- | --- | --- | --- | --- | --- | --- | --- | --- |
|  | M3 | | M4 | | | M5 | | | M3 | | M4 | | | M5 |  |
| Time(s) | 44.96 | | 51.28 | | | 48.08 | | | 20.90 | 20.20 | | | | 20.33 |  |
| Method | | GA | | | GA-HMSO | | | MCTS_100 | | | | MCTS-HMSO_100 | | | |
| Time(s) | | 33.28 | | 24.48 | | | 417.79 | | | | | | 719.86 | | |

**C-4.** The current guidance is purely sequence-based and evolutionary. It does not explicitly incorporate physics-based energy functions or detailed structural models. The authors should discuss the potential and challenges of integrating with explicit structural or physical constraints.

**R-4.** We appreciate this constructive suggestion. In the revised manuscript, we added a discussion section. First, ProtHMSO establishes an efficient search mechanism by leveraging the rich evolutionary priors in ProtLMs, effectively validating the rationality of implicit sequence constraints. While integrating physics-based energy functions can further enhance structural stability, a key challenge remains: the trade-off between computational efficiency and search depth. The high computational overhead of atomic-level physics calculations conflicts with ProtHMSO's core advantage of rapidly traversing vast combinatorial spaces. Therefore, a "coarse-to-fine" hybrid strategy is the most feasible path for future optimization.

**To Reviewer #3**

**C-overall**: This paper presents a heuristic multi-site optimization framework that integrates masked protein language models with evolutionary algorithms for protein sequence design. This paper successfully merges the contextual prediction power of masked language models with heuristic search strategies, enabling more biologically plausible and efficient exploration of protein sequence space. The proposed method is designed not only as a standalone optimizer but also as a plug-and-play module that enhances existing methods such as genetic algorithms and monte carlo tree search. This modularity increases its applicability and potential impact in computational protein design. The extensive experiments on two datasets provide a comprehensive evaluation.

This paper presents a well-designed framework and is clearly written, but there are still some limitations to be addressed before published.

**R-overall:** Thank you for your meticulous review of our manuscript. We have revised the manuscript extensively according to your valuable suggestions.

**C-1.** While the method is shown to be effective, an analysis of its computational cost relative to other deep learning–based design methods is absent. A discussion or benchmark comparing wall-clock time and/or FLOPs against other model-guided optimizers would help users assess the trade-off between performance and cost.

**R-1.** For a discussion on this part, please refer to the response to R-3 in Reviewer #2.

**C-2**. The framework introduces several important hyperparameters, such as mutation probability schedules, top-k substitutions, group scoring in MCTS-HMSO. A rigorous justification is needed to demonstrate that the presented configuration is optimal or robust.

**R-2.** Based on your suggestion, we conducted supplementary experiments regarding the parameter settings for mutation probability schedules, top-k substitutions, and group scoring in MCTS-HMSO. Details are as follows:

To determine the optimal configuration for mutation probability parameters within GA, we systematically evaluated the impact of iteration thresholds and various mutation probability strategies on the predicted probability distribution of generated sequences. First, regarding the selection of the iteration threshold, we compared it performance with thresholds set at 5, 7, 10, and 15 (as shown in Fig S2). The results indicate that an iteration threshold of 5 yields the highest mean probability for AMPs (Mean: 0.90), while maintaining a favorable balance in the probability distribution related to MIC. As the threshold increases to 10 or 15, although some samples retain high prediction scores, the overall mean of the distribution shows a downward trend, accompanied by an increase in dispersion. Consequently, we fixed the iteration threshold at 5 for subsequent experiments to ensure the algorithm could rapidly converge to a high-quality solution space in the early stages.

**Fig S2. Probability value distributions for different iteration thresholds.** The x-axis labels indicate the specific iteration threshold (5, 7, 10, or 15) applied in the GA-HMSO algorithm for AMP and MIC predictions.

Building on this, we further investigated the influence of mutation probability strategies on model performance by comparing three modes: fixed low probability, fixed high probability, and dynamic probability. Results in Fig S3 reveal that the fixed low probability strategy (GA-HMSO-low) limits the diversity of generated sequences, resulting in a lower overall mean probability (AMP Mean: 0.29). Conversely, while the fixed high probability strategy enhances exploration, it risks disrupting established superior structures. In contrast, the dynamic probability strategy (GA-HMSO-dynamic) demonstrates the best comprehensive performance, achieving an AMP probability mean of 0.90 while maintaining a high MIC-related probability (Mean: 0.83). This dynamic adjustment mechanism effectively balances the algorithm's global exploration and local exploitation capabilities, thereby generating sequences with higher predicted activity and superior property distributions.

Fig S3. **Comparison of probability distributions across varying mutation settings.** The x-axis labels denote the specific mutation strategy applied: *fixed* uses a constant probability, *dynamic* employs an adaptive probability, while *high* and *low* represent mutation probabilities maintained at consistently high or low levels throughout the process.

For a discussion on top *k*, please refer to reply R-2 in Review #1.

In the design of the group scoring mechanism for the MCTS-HMSO algorithm, we evaluated two distinct strategies to determine the optimal method for assessing the performance of nodes within a group: selecting the maximum UCB value of visited nodes within the group *max*, and calculating the weighted average of visited nodes *sum*. To verify the efficacy of these strategies, we conducted experiments across varying numbers of iterations (100, 300, and 500) and analyzed the resulting probability distributions for AMP and MIC (as shown in the Fig S4).

**Fig S4. Comparison of group scoring mechanisms and iteration counts in the MCTS-HMSO algorithm.** ***max*** represents the group score using the maximum value of explored nodes within the group, while ***sum*** utilizes the weighted sum of the explored nodes. The numerical values (100, 300, 500) denote the number of iterations performed.

The experimental results demonstrate that the performance of these two scoring strategies is highly comparable across different iteration stages. Regardless of whether the iteration count was 100, 300, or 500, there were no significant differences in the shape or the mean values of the probability distributions generated by the "max" and "sum" strategies. For instance, at 500 iterations, the *max* strategy yielded a mean AMP probability of 0.96 and a mean MIC probability of 0.66, which were identical to those obtained using the *sum* strategy. This suggests that the algorithm possesses a degree of robustness regarding the specific aggregation method used for group scoring. However, considering that the weighted average provides a more comprehensive reflection of the overall quality of all explored nodes within a group, rather than relying solely on a single optimal node, thereby potentially offering more stable guidance. We ultimately selected the weighted average as the standard method for group scoring in this study.

**C-3.** While conserved regions and structural changes are analyzed, this paper lacks to leverage the ESM-2 model to offer deeper biological insights. For instance, analyzing the model's attention patterns or constructing simple sequence-structure mapping around predicted mutation sites could help answer why certain sites are targeted. This would move the analysis beyond correlation to provide testable hypotheses about functional impact.

**R-3.** To investigate the rationality of mutation site selection by the ESM2 model, we analyzed the highest (Top) and lowest (Bottom) probability mutants of the sequence `KVPIGAIKKGGKIIKKGLGVIGAAGTAHEVYSHVKNRH' in Fig S5 in Supplementary Information.

Fig S5. Comparative analysis of secondary structures for the original sequence and ProtHMSO-predicted mutants. (a) Two-dimensional structure of the original sequence. (b) Two-dimensional structure of the sequence with the lowest mutation probability. (c) Two-dimensional structure of the sequence with the highest mutation probability.

The Top sequence demonstrates a strong tendency to optimize AMP characteristics. It eliminates helix-breaking proline residues to enhance conformational stability and incorporates lysine mutations (e.g., N34K) to increase the net positive charge, thereby facilitating electrostatic interactions with negatively charged bacterial membranes. Furthermore, the sequence retains core hydrophobic segments, preserving the amphipathic $\alpha$-helical architecture essential for membrane insertion and pore formation.

Conversely, the Bottom sequence reflects the model's sensitivity to deleterious mutations. It introduces cysteines, which pose a risk of non-specific disulfide aggregation, and accumulates bulky aromatic residues (Tyrosine, Tryptophan) that cause excessive hydrophobicity and steric hindrance. Consequently, the characteristic $\alpha$-helical structure completely degenerates into a random coil, disrupting the amphipathic balance required for function. This stark contrast confirms that ESM2 has effectively internalized the complex mapping between sequence patterns, structural stability, and physicochemical properties.

**C-4.** .In conclusion, this paper briefly mentions future integration with physics-based energy functions. Given the growing trend of hybrid ML-physics approaches, expanding this point into a concrete discussion within the main text would be valuable.

**R-4.** We have expanded the discussion to explicitly address the integration of physics-based energy functions. Specifically, we analyze the distinction between the evolutionary plausibility captured by ProtHMSO and rigorous thermodynamic stability, proposing that physical models (e.g., Rosetta or MD) are essential for validating structural integrity. To resolve the conflict between computational overhead and search efficiency, we outline a "coarse-to-fine" hybrid strategy: utilizing ProtHMSO as a high-throughput screener to navigate the fitness landscape, followed by physics-based scoring for fine-grained validation, thereby balancing the exploration speed of deep learning with the physical accuracy of traditional biophysics.
