# Supplementary material for: Heuristic multi-site optimization for protein sequence design using Masked Protein Language Models
Source: PLoS Comput Biol. 2026 Jun 5;22(6):e1014365. doi: 10.1371/journal.pcbi.1014365 (PMC13252849; doi:10.1371/journal.pcbi.1014365)
Supplement: Attachment — Submitted filename: Response Letter for ProtHMSO-updata.docx [file pcbi.1014365.s004.docx]

Dear editor,

Thanks for your feedback and the attached review comments from the reviewers. Their constructive comments are very helpful for further strengthening the presentation of our paper. As suggested, the manuscript has been carefully revised. Our point-to-point responses can be summarized as follows. For clarity, the reviewers’ comments are colored in green beginning with **C** (comment), while our responses in blue beginning with **R** (response). All the revised texts in the manuscript are highlighted with red.

**To Reviewer #1**

**C-overall**: This paper proposed a heuristic multi-site optimization framework ProtHMSO, that leverages masked protein language models (ProtLMs) for context-aware sequence exploration. ProtHMSO is further applied to replace the exploration strategies in genetic algorithms and Monte Carlo tree search for improving their convergence efficiency. Benchmark experiments demonstrate that protein sequences generated by ProtHMSO exhibit superior functional performance and closer alignment with natural sequence distribution.

**R-overall:** Thank you for your careful review with regard to our manuscript. We have made comprehensive changes according to your suggestions.

**C-1.** The proposed framework integrates masked protein language models with heuristic multi-site optimization, the degree of methodological novelty remains somewhat unclear. The use of ProtLM-derived substitution probabilities as evolutionary priors appears conceptually related to existing PLM-guided protein design strategies and evolutionary-constrained search approaches. It would benefit for this paper from a clearer demonstrate of the key methodological distinctions between ProtHMSO and prior PLM-guided sequence optimization methods, particularly in terms of search dynamics, constraint formulation, and theoretical justification of the heuristic strategy.

**R-1.** We are very grateful for the valuable comments from the reviewers. We fully understand your focus on the methodological novelty and the distinction from existing protein language model-based strategies. We have revised the last paragraph of the introduction, the description of the HMSO method, and the conclusion to further emphasize the innovativeness of our method. In fact, ProtHMSO is a contemporaneous development with current PLM-guided protein design methods, such as the MULTI-evolve framework recently published in *Science* (*Rapid directed evolution guided by protein language models and epistatic interactions*). The core paradigm of MULTI-evolve is to first utilize the zero-sample capability of PLM to screen for single-point mutations, then rely on experimental methods to construct and test a double-mutant dataset, and finally train a supervised fully connected neural network to learn epistatic effects and predict multi-point mutations. In contrast, ProtHMSO, in addressing the core challenge of multi-point mutations, does not collect intermediate experimental data or conduct additional downstream model training. Our core difference lies in the fact that ProtHMSO directly leverages the rich epistatic effects and co-evolutionary information implicit in the deep architecture of Masked Protein Language Models (such as ESM-2) to guide multi-site co-mutation in a completely zero-shot manner. ProtHMSO's heuristic strategy is not simply the summing of single-point probabilities, but rather uses a dynamic masking mechanism to take the entire sequence context as conditional input in each iteration. This means that when a mutation occurs at one site, the model updates the substitution probabilities of other sites in real time, naturally forming a dynamic, context-aware evolutionary constraint within a vast combinatorial search space. This dynamic search ensures that multi-site mutations, while introducing new features, strictly adhere to the prior laws of biophysics and structural stability, avoiding the structural damage that traditional methods often cause in multi-site mutations, and exhibiting a more elegant, efficient, and purely computationally driven design loop.

Furthermore, ProtHMSO is not merely an isolated sequence generation tool, but a highly versatile and scalable plug-and-play heuristic enhancement module. In traditional computational protein design, while classic evolutionary algorithms such as genetic algorithms and Monte Carlo tree search possess global search capabilities, their reliance on blind random mutations often results in extremely low exploration efficiency when faced with the exponentially expanding high-dimensional combinatorial space of protein sequences. These algorithms are also prone to getting trapped in local optima or generating a large number of invalid sequences. This study creatively integrates the heuristic strategy of ProtHMSO deeply into the underlying logic of GA and MCTS, fundamentally reshaping the search dynamics of these classic algorithms. In GA-HMSO, we replace the traditional random mutation operator with the evolutionary prior of ProtLM, ensuring that each reproduction of the population evolves along a high-potential fitness landscape. In MCTS-HMSO, we transform ProtHMSO into a node selection guide for the tree search expansion stage, using the probability matrix output by the model to heuristically prune the massive number of child nodes, guiding the search tree to explore deeply along mutation paths with synergistic effects. This strategy of coupling the context-aware capabilities of cutting-edge deep learning language models with the global optimization framework of classical operations research and heuristic algorithms at a low level is unprecedented in the existing literature. It not only significantly reduces the ineffective search space and greatly improves the convergence efficiency of the algorithm, but also demonstrates the broad applicability and modular potential of this framework in the entire field of computational protein design. This is a key innovation that distinguishes it from other rigid end-to-end deep learning design methods.

**C-2.** The experimental evaluation demonstrates improved functional performance; however, it is unclear whether the benchmarks sufficiently cover diverse protein families and functional regimes. Since protein sequence design methods may overfit to specific fitness landscapes or predictive models, further validation across heterogeneous datasets and experimentally validated fitness landscapes would strengthen the generalizability claims. Additionally, it would be valuable to assess robustness under varying fitness model qualities or noisy predictors.

**R-2.** Thank you for your suggestion. It was with the same consideration in mind that we specifically introduced two distinct and highly heterogeneous benchmark datasets in the experimental design phase of this study (page 11 of the paper, "Experiments"): the antimicrobial peptide (AMP) dataset and the ProteinGym database, which encompasses large-scale real-world experimental data. Below is a detailed explanation of the datasets used and their specific partitioning methods:

First, to evaluate the model's multi-objective optimization capability for short-sequence peptides in specific functional scenarios, we used the antimicrobial peptide dataset. This dataset, derived from the DBAASP database, retained 4,505 unique active sequences after rigorous deduplication and filtering. To comprehensively test the algorithm's optimization capabilities under different initial fitness conditions, we did not test the dataset as a whole. Instead, based on the initial antimicrobial probability ($P_{AMP}$) and minimum inhibitory concentration probability ($P_{MIC}$) of the sequences, we finely divided them into three different optimization scenarios with a threshold of 0.8: Case1 (Activity Enhancement Scenario, 875 sequences): Initially, the function is good ($P_{AMP}$> 0.8) but the activity is insufficient ($P_{MIC}$< 0.8), requiring the model to improve activity while maintaining function; Case2 (Function Enhancement Scenario, 184 sequences): Initially, the activity is good ($P_{MIC}$>0.8) but the functionality is insufficient ($P_{AMP}$ <0.8), focusing on optimizing functionality; Case3 (High-Challenge Scenario, 476 sequences): Initially, both function and activity are poor ($P_{AMP}$< 0.8 and $P_{MIC}$ <0.8). The most stringent test, with a score of 0.8, requires the model to achieve significant improvements in both objectives simultaneously across complex environments.

Secondly, unlike short peptide datasets that primarily rely on predictive model scores, ProteinGym contains an extremely rich family of long-sequence proteins, and its evaluation criteria are entirely based on real biological experiments or clinical annotations: the Clinical Benchmark includes over 63,000 mutants from 2,525 different genes (with an average sequence length of up to 709 amino acids), ensuring a high degree of diversity in protein families and structures within the test set. Its fitness labels are based on real clinical annotations (1 representing pathogenicity, 0 representing benignity). We tested on this dataset whether ProtHMSO could stably prioritize generating functionally benign (non-pathogenic) mutant sequences across different protein families; the DMS Benchmark includes 217 independent assays covering over 2.4 million mutants. The fitness score (DMS_score) here is not derived from any machine learning predictive model, but rather from continuous, real-world experimental measurements. To further explore the effectiveness of multi-site optimization, we further divided the dataset into single-site and two-site subsets. We used ProtHMSO to heuristically explore the conserved functional domains of these sequences and compared the overlap between the generated mutants and the top 10, 20, and 50 variants in real-world experiments. The results show that ProtHMSO can highly enrich high-fitness variants from real-world experiments.

Regarding with the robustness of our evaluation method under noisy predictors, we specifically selected the most challenging and representative Case 3 dataset for supplementary experiments. In the specific experimental setup, we added Gaussian noise with a mean of 0 and a standard deviation of 0.05 onto the prediction probability results of the original AMP and MIC classification models, and strictly limited the final scores to a reasonable range of [0, 1] to simulate a fitness model with noise. The experimental results (as shown in Fig R4-1) indicate that, with the introduction of noise interference (groups marked "_N" in the figure), the score distribution and mean of each model variant on the function enhancement and activity enhancement tasks showed a smaller fluctuation compared to the original noise-free model (for example, the mean of the best-performing ProtHMSO_M5 model slightly decreased from 0.86 to 0.85 in the function enhancement task and from 0.56 to 0.55 in the activity enhancement task). This result demonstrates that our method does exhibit some fluctuation when facing fitness models of different qualities or with noise, but the degree of fluctuation is relatively small.

Fig R4-1. Comparison of AMP and MIC probability values of different methods. *ProtHMSO* indicates a variant generated using ESM-2, *N* indicates a variant generated after adding noise to the predictor, *M1* indicates a mutation at one site, and so on.

**C-3.** ProtHMSO incorporates evolutionary priors to limit the search space, but this paper does not clarify whether structural stability or folding consistency is explicitly evaluated. Multi-site mutations can introduce epistatic effects that destabilize native conformations. Including structural validation metrics (e.g., predicted folding stability, structural deviation, or AlphaFold confidence scores) would provide stronger evidence that the generated variants maintain biophysical plausibility alongside improved fitness.

**R-3.** In the original manuscript, we already introduced structural validation metrics. In the "Metrics" section and "Table 1," we used the Local Distance Difference Test (pLDDT) score based on ESMFold predictions to evaluate the structural confidence and validity of the generated sequences. The results preliminarily show that the variants generated by ProtHMSO maintain structural stability better than random mutations. To further validate the biophysical properties of the generated variants, we specifically supplemented the experiment with explicit structure and energy assessments for the most challenging and performance-reflecting *Case3* dataset (which requires simultaneous optimization of function and activity). In the specific experimental procedure, we first used AlphaFold to predict the three-dimensional structures of the original sequence and variants generated by different mutation strategies, and then used PyRosetta to calculate the precise energy values of these predicted conformations. Generally, for short peptide molecules, an energy value between -10 and 5 is considered a usable conformation with reasonable biophysical properties and stable structure. As shown in Fig R4-2, the energy distribution of the ProtHMSO-generated variants largely falls within the normal and reasonable range of [-10, 5]. Furthermore, with the increase in the number of mutation sites (from M1 to M5), the ProtHMSO-guided mutants exhibit a lower (i.e., more stable) average energy value compared to random mutations. For example, with 5 mutation sites (M5), the average energy value of the ProtHMSO variant drops to 3.34, significantly better than random mutations under the same conditions (average 4.91) and even better than the original sequence in Case 3 (average 5.06). This result demonstrates that ProtHMSO, leveraging the evolutionary priors inherent in the language model, can effectively overcome the epistatic effects caused by multi-site mutations, significantly improving sequence fitness (function and activity) while maintaining or even optimizing the thermodynamic stability of its native conformation.

Fig R4-2. Comparison of AMP and MIC probability values of different methods. *Case3* represents the energy value distribution of the original dataset, *Random* represents the energy value distribution of the variant generated using random mutations, *ProtHMSO* represents the energy value distribution of the variant generated using ESM-2, M1 represents a mutation at one site, and so on.
