# Supplementary material for: Heuristic multi-site optimization for protein sequence design using Masked Protein Language Models
Source: PLoS Comput Biol. 2026 Jun 5;22(6):e1014365. doi: 10.1371/journal.pcbi.1014365 (PMC13252849; doi:10.1371/journal.pcbi.1014365)
Supplement: Attachment — Submitted filename: Response Letter for ProtHMSO-R3.docx [file pcbi.1014365.s005.docx]

Dear editor,

Thanks for your feedback and the attached review comments from the reviewer. The constructive comments are very helpful for further strengthening the presentation of our paper. As suggested, the manuscript has been carefully revised. Our point-to-point responses can be summarized as follows. For clarity, the reviewers’ comments are colored in green beginning with **C** (comment), while our responses in blue beginning with **R** (response). All the revised texts in the manuscript are highlighted with red.

**To Reviewer #1**

**C-overall**: The paper mentions that ProtHMSO can implicitly capture synergistic inter-residue epistasis zero-shot, but lacks a concrete explanation of the underlying principles. It is recommended to add a brief theoretical explanation to the Methodology or Discussion section.

**R-overall:** Thank you for your careful review with regard to our manuscript. We have made comprehensive changes according to your suggestions.

**C-1.** The paper mentions that ProtHMSO can implicitly capture synergistic inter-residue epistasis zero-shot, but lacks a concrete explanation of the underlying principles. It is recommended to add a brief theoretical explanation to the Methodology or Discussion section.

**R-1.** We are very grateful for your valuable comments. To address this, we have expanded the *Heuristic Multi-site Optimization Framework* subsection in the Methodology. Specifically, we explained that masked protein language models (like ESM-2) learn the joint probability distribution of natural protein sequences through the multi-head self-attention mechanism during pre-training. Mathematically, the substitution probability at any given site 𝑖 is computed as a conditional probability $p(x_{i}|X_{\backslash i})$, which dynamically depends on the global sequence context. Therefore, if a mutation occurs at site 𝑗, the attention weights and the resulting probability distribution at site 𝑖 will inherently shift. This dynamic contextual dependence allows the model to implicitly evaluate non-linear interactions (epistasis) between distant residues without requiring any task-specific supervised training (zero-shot) or explicit structural annotations.

**C-2.** ProtHMSO is further applied to replace the exploration strategies in genetic algorithms (GAs) and Monte Carlo tree search (MCTS) for improving their convergence efficiency. The default optimization strategies are modified. GA-HMSO adopts a dynamic crossover and mutation rate, while MCTS-HMSO employs group-based UCB values. It is suggested to clarify the underlying philosophy behind these modifications.

**R-2.** We completely agree that clarifying the underlying philosophy behind these algorithmic modifications is crucial for readers to understand the design of our framework. Following your suggestion, we have explicitly added the explanations into the *Methodology* section of the revised manuscript. For GA-HMSO, the clarification has been added to the *Heuristic Genetic Algorithm based on HMSO* section. For MCTS-HMSO, the clarification has been added to the *Heuristic Monte Carlo Tree Search based on HMSO* section.

1. The philosophy behind the dynamic crossover and mutation rate in GA-HMSO:

The primary motivation is to optimally balance global exploration and local exploration across the highly rugged protein fitness landscape. In the early stages of evolution, a high mutation rate is employed to encourage broad exploration of the vast sequence space, effectively preventing the population from being trapped in local optima. As the evolution progresses, the algorithm needs to converge. Therefore, the mutation rate dynamically decreases to protect the high-fitness structural motifs (schemas) that have already been discovered, allowing the algorithm to fine-tune the sequences and refine the local optimization.

2. The philosophy behind the group-based UCB values in MCTS-HMSO:

The core motivation here is to mitigate the severe combinatorial explosion inherent in multi-site protein design. If standard MCTS is used, every possible amino acid substitution at every site is treated as an independent branch, leading to an excessively wide and shallow search tree. By grouping sub-nodes based on their mutation positions, we introduce a two-step decision-making process. The algorithm first evaluates which specific residue site is most promising to mutate (macro-exploration via group scores), and then selects the best specific amino acid substitution at that site (micro-exploitation via individual UCB values). This strategy significantly prunes the search space and focuses the computational budget on the most impactful structural regions.

**C-3.** The literature review should be updated to cover the latest works in the field, such as the diffusion-based protein sequence generation methods (EvoDiff) and reinforcement learning-based methods for fine-tuning protein language models.

**R-3.** We sincerely thank the reviewer for pointing out these important and highly relevant recent advancements. Based on your valuable suggestions, we have added some new research findings to the *Introduction* section of the revised manuscript. This mainly includes introductions to DFdiffusion, ProteinMPNN, EvoDiff, and reinforcement learning.

**C-4.** This paper also needs to clarify the practical application scenarios and transformation potential, such as how to accelerate the protein design process.

**R-4.** We sincerely thank your insightful suggestion, which helps bridge the gap between our computational framework and real-world utility. In response, we have added a dedicated paragraph in the *Discussion* section of the revised manuscript to explicitly clarify the practical application scenarios and transformation potential of ProtHMSO. Specifically, we elaborated that ProtHMSO can be directly applied to the optimization of therapeutic peptides, enzymes, and antibodies by replacing blind mutagenesis in traditional directed evolution. We further explained that it accelerates the protein design process through two perspectives: computationally, it utilizes prior knowledge for heuristic search, significantly reducing the combinatorial search space and enabling the selection of documents to be reduced from millions to only tens or even hundreds of variations; experimentally, it improves the conversion success rate in wet laboratories by enhancing the antimicrobial activity and antimicrobial capacity of antimicrobial peptides. By integrating into existing workflows as a plug-and-play module, ProtHMSO significantly increases the experimental hit rate and reduces the costs of AMP synthesis and high-throughput screening, thereby demonstrating strong transformation potential for accelerating real-world protein discovery.
