# Supplementary material for: Heuristic multi-site optimization for protein sequence design using Masked Protein Language Models
Source: PLoS Comput Biol. 2026 Jun 5;22(6):e1014365. doi: 10.1371/journal.pcbi.1014365 (PMC13252849; doi:10.1371/journal.pcbi.1014365)
Supplement: Peer review history

# Peer review: Decision Letter 0

**DOI:** 10.1371/journal.pcbi.1014365.r001

**Authors:** Yanbu Guo

23 Dec 2025

PCOMPBIOL-D-25-01541

Heuristic Multi-site Optimization for Protein Sequence Design using Masked Protein Language Models

PLOS Computational Biology

Dear Dr. Chen,

Thank you for submitting your manuscript to PLOS Computational Biology. After careful consideration, we feel that it has merit but does not fully meet PLOS Computational Biology's publication criteria as it currently stands. Therefore, we invite you to submit a revised version of the manuscript that addresses the points raised during the review process.

Please submit your revised manuscript by Feb 22 2026 11:59PM. If you will need more time than this to complete your revisions, please reply to this message or contact the journal office at ploscompbiol@plos.org. When you're ready to submit your revision, log on to https://www.editorialmanager.com/pcompbiol/ and select the 'Submissions Needing Revision' folder to locate your manuscript file.

Please include the following items when submitting your revised manuscript:

* A letter that responds to each point raised by the editor and reviewer(s). You should upload this letter as a separate file labeled 'Response to Reviewers'. This file does not need to include responses to formatting updates and technical items listed in the 'Journal Requirements' section below.

* A marked-up copy of your manuscript that highlights changes made to the original version. You should upload this as a separate file labeled 'Revised Manuscript with Track Changes'.

* An unmarked version of your revised paper without tracked changes. You should upload this as a separate file labeled 'Manuscript'.

If you would like to make changes to your financial disclosure, competing interests statement, or data availability statement, please make these updates within the submission form at the time of resubmission. Guidelines for resubmitting your figure files are available below the reviewer comments at the end of this letter

We look forward to receiving your revised manuscript.

Kind regards,

Yanbu Guo, Ph.D.

Academic Editor

PLOS Computational Biology

Shihua Zhang

Section Editor

PLOS Computational Biology

**Journal Requirements:**

1) Please ensure that the CRediT author contributions listed for every co-author are completed accurately and in full.

At this stage, the following Authors/Authors require contributions: Lijuan Wang, Yuze Wang, Chen Qiu, Liwei Xiao, Xianliang Liu, and Junjie Chen. Please ensure that the full contributions of each author are acknowledged in the "Add/Edit/Remove Authors" section of our submission form.

The list of CRediT author contributions may be found here: https://journals.plos.org/ploscompbiol/s/authorship#loc-author-contributions

2) We ask that a manuscript source file is provided at Revision. Please upload your manuscript file as a .doc, .docx, .rtf or .tex. If you are providing a .tex file, please upload it under the item type u2018LaTeX Source Fileu2019 and leave your .pdf version as the item type u2018Manuscriptu2019.

3) Please upload all main figures as separate Figure files in .tif or .eps format. For more information about how to convert and format your figure files please see our guidelines:

https://journals.plos.org/ploscompbiol/s/figures

4) Please amend your detailed Financial Disclosure statement. This is published with the article. It must therefore be completed in full sentences and contain the exact wording you wish to be published.

- State the initials, alongside each funding source, of each author to receive each grant. For example: "This work was supported by the National Institutes of Health (####### to AM; ###### to CJ) and the National Science Foundation (###### to AM)."

- State what role the funders took in the study. If the funders had no role in your study, please state: "The funders had no role in study design, data collection and analysis, decision to publish, or preparation of the manuscript.".

If you did not receive any funding for this study, please simply state: u201cThe authors received no specific funding for this work.u201d

**Reviewers' comments:**

Reviewer's Responses to Questions

**Comments to the Authors:**

**Please note here if the review is uploaded as an attachment.**

Reviewer #1: This paper proposed ProtHMSO, a heuristic multi-site optimization framework. ProtHMSO mimics natural evolutionary mechanism by employing protein language models (ProtLM)-derived substitution probabilities to heuristically search for rational mutations. ProtHMSO can also be integrated into GA and MCTS algorithms to improve their optimization performance. Benchmark experiments demonstrate that ProtHMSO exhibits a SOTA performance compared with baseline methods. This paper is novel, providing an efficient strategy for protein sequence optimization.

Strengths:

1. Introduce a heuristic multi-site optimization framework to guide the selection of multi-site mutations, breaking through the limitation of traditional single-point mutations that ignore epistatic interactions between sites.

2. Integrate into the genetic algorithm (GA) and Monte Carlo tree search (MCTS), enhancing their convergence efficiency.

3. Achieve a SOTA performance on multiple datasets, verifying its effectiveness in different lengths and scenarios.

Minor weaknesses:

1. The challenge faced by existing multi-site optimization should be highlighted, and the motivation of this work can be further emphasized in the introduction.

2. It is better to analyze the impact of the k value in top-k selection in Algorithm 1.

Reviewer #2: This paper proposed a heuristic multi-site optimization framework, ProtHMSO, for protein sequence design. Its key innovation lies in leveraging evolutionary priors from masked language models (ESM-2) to guide the search process, effectively overcoming the limitations of conventional blind search methods. ProtHMSO can be used as standalone for directed optimization and as a plugin to enhance GAs and MCTS. Experiments on AMP dataset and ProteinGym dataset show that ProtHMSO achieves faster convergence and better results on benchmark datasets, and it is especially effective in challenging cases where both activity and functionality needed improvement.

The manuscript is novel and well-written. But there are still some minor issues to be addressed.

1.The protein language models, genetic algorithm and Monte Carlo tree search have been widely used in various fields. Therefore, their introduction in related work section could be more concise.

2.The AMP data was split into four disjoint subsets, but the reason how to choose the thresholds is unclear.

3.While ProtHMSO achieves faster convergence, running inference on a large model like ESM-2 (650M) for every mutation step is computationally intensive. The hardware and total cost time should be given to help the users evaluate the practical trade-offs of using a large model in specific scenarios.

4.The current guidance is purely sequence-based and evolutionary. It does not explicitly incorporate physics-based energy functions or detailed structural models. The authors should discuss the potential and challenges of integrating with explicit structural or physical constraints.

Reviewer #3: This paper presents a heuristic multi-site optimization framework that integrates masked protein language models with evolutionary algorithms for protein sequence design. This paper successfully merges the contextual prediction power of masked language models with heuristic search strategies, enabling more biologically plausible and efficient exploration of protein sequence space. The proposed method is designed not only as a standalone optimizer but also as a plug-and-play module that enhances existing methods such as genetic algorithms and monte carlo tree search. This modularity increases its applicability and potential impact in computational protein design. The extensive experiments on two datasets provide a comprehensive evaluation.

This paper presents a well-designed framework and is clearly written, but there are still some limitations to be addressed before published.

1.While the method is shown to be effective, an analysis of its computational cost relative to other deep learning–based design methods is absent. A discussion or benchmark comparing wall-clock time and/or FLOPs against other model-guided optimizers would help users assess the trade-off between performance and cost.

2.The framework introduces several important hyperparameters, such as mutation probability schedules, top-k substitutions, group scoring in MCTS-HMSO. A rigorous justification is needed to demonstrate that the presented configuration is optimal or robust.

3.While conserved regions and structural changes are analyzed, this paper lacks to leverage the ESM-2 model to offer deeper biological insights. For instance, analyzing the model's attention patterns or constructing simple sequence-structure mapping around predicted mutation sites could help answer why certain sites are targeted. This would move the analysis beyond correlation to provide testable hypotheses about functional impact.

4.In conclusion, this paper briefly mentions future integration with physics-based energy functions. Given the growing trend of hybrid ML-physics approaches, expanding this point into a concrete discussion within the main text would be valuable.

**********

**Have the authors made all data and (if applicable) computational code underlying the findings in their manuscript fully available?**

The PLOS Data policy requires authors to make all data and code underlying the findings described in their manuscript fully available without restriction, with rare exception (please refer to the Data Availability Statement in the manuscript PDF file). The data and code should be provided as part of the manuscript or its supporting information, or deposited to a public repository. For example, in addition to summary statistics, the data points behind means, medians and variance measures should be available. If there are restrictions on publicly sharing data or code —e.g. participant privacy or use of data from a third party—those must be specified.

Reviewer #1: None

Reviewer #2: Yes

Reviewer #3: Yes

**********

PLOS authors have the option to publish the peer review history of their article (what does this mean?). If published, this will include your full peer review and any attached files.

If you choose “no”, your identity will remain anonymous but your review may still be made public.

**Do you want your identity to be public for this peer review?**   For information about this choice, including consent withdrawal, please see our Privacy Policy.

Reviewer #1: No

Reviewer #2: No

Reviewer #3: **Yes**

[NOTE: If reviewer comments were submitted as an attachment file, they will be attached to this email and accessible via the submission site. Please log into your account, locate the manuscript record, and check for the action link "View Attachments". If this link does not appear, there are no attachment files.]

**Figure resubmission:**

While revising your submission, we strongly recommend that you use PLOS’s NAAS tool (https://ngplosjournals.pagemajik.ai/artanalysis) to test your figure files. NAAS can convert your figure files to the TIFF file type and meet basic requirements (such as print size, resolution), or provide you with a report on issues that do not meet our requirements and that NAAS cannot fix.

After uploading your figures to PLOS’s NAAS tool - https://ngplosjournals.pagemajik.ai/artanalysis, NAAS will process the files provided and display the results in the "Uploaded Files" section of the page as the processing is complete. If the uploaded figures meet our requirements (or NAAS is able to fix the files to meet our requirements), the figure will be marked as "fixed" above. If NAAS is unable to fix the files, a red "failed" label will appear above. When NAAS has confirmed that the figure files meet our requirements, please download the file via the download option, and include these NAAS processed figure files when submitting your revised manuscript.

**Reproducibility:**

To enhance the reproducibility of your results, we recommend that authors of applicable studies deposit laboratory protocols in protocols.io, where a protocol can be assigned its own identifier (DOI) such that it can be cited independently in the future. Additionally, PLOS ONE offers an option to publish peer-reviewed clinical study protocols. Read more information on sharing protocols at https://plos.org/protocols?utm_medium=editorial-email&utm_source=authorletters&utm_campaign=protocols

# Author comment: Author response to Decision Letter 1

**DOI:** 10.1371/journal.pcbi.1014365.r002

11 Feb 2026

Attachment Submitted filename: Response Letter for ProtHMSO.docx

# Peer review: Decision Letter 1

**DOI:** 10.1371/journal.pcbi.1014365.r003

**Authors:** Yanbu Guo

10 Mar 2026

PCOMPBIOL-D-25-01541R1

Heuristic Multi-site Optimization for Protein Sequence Design using Masked Protein Language Models

PLOS Computational Biology

Dear Dr. Chen,

Thank you for submitting your manuscript to PLOS Computational Biology. After careful consideration, we feel that it has merit but does not fully meet PLOS Computational Biology's publication criteria as it currently stands. Therefore, we invite you to submit a revised version of the manuscript that addresses the points raised during the review process.

Please submit your revised manuscript by May 10 2026 11:59PM. If you will need more time than this to complete your revisions, please reply to this message or contact the journal office at ploscompbiol@plos.org. When you're ready to submit your revision, log on to https://www.editorialmanager.com/pcompbiol/ and select the 'Submissions Needing Revision' folder to locate your manuscript file.

Please include the following items when submitting your revised manuscript:

* A letter that responds to each point raised by the editor and reviewer(s). You should upload this letter as a separate file labeled 'Response to Reviewers'. This file does not need to include responses to formatting updates and technical items listed in the 'Journal Requirements' section below.

* A marked-up copy of your manuscript that highlights changes made to the original version. You should upload this as a separate file labeled 'Revised Manuscript with Track Changes'.

* An unmarked version of your revised paper without tracked changes. You should upload this as a separate file labeled 'Manuscript'.

If you would like to make changes to your financial disclosure, competing interests statement, or data availability statement, please make these updates within the submission form at the time of resubmission. Guidelines for resubmitting your figure files are available below the reviewer comments at the end of this letter.

We look forward to receiving your revised manuscript.

Kind regards,

Yanbu Guo, Ph.D.

Academic Editor

PLOS Computational Biology

Shihua Zhang

Section Editor

PLOS Computational Biology

Note: If the reviewer comments include a recommendation to cite specific previously published works, please review and evaluate these publications to determine whether they are relevant and should be cited. There is no requirement to cite these works unless the editor has indicated otherwise.

**Reviewers' comments:**

Reviewer's Responses to Questions

Reviewer #1: The authors have addressed all of my concerns raised during the review process.

Reviewer #3: All questions and comments were satisfactorily addressed.

Reviewer #4: This paper proposed a heuristic multi-site optimization framework ProtHMSO, that leverages masked protein language models (ProtLMs) for context-aware sequence exploration. ProtHMSO is further applied to replace the exploration strategies in genetic algorithms and Monte Carlo tree search for improving their convergence efficiency. Benchmark experiments demonstrate that protein sequences generated by ProtHMSO exhibit superior functional performance and closer alignment with natural sequence distribution. However, I have some concerns below.

1. The proposed framework integrates masked protein language models with heuristic multi-site optimization, the degree of methodological novelty remains somewhat unclear. The use of ProtLM-derived substitution probabilities as evolutionary priors appears conceptually related to existing PLM-guided protein design strategies and evolutionary-constrained search approaches. It would benefit for this paper from a clearer demonstrate of the key methodological distinctions between ProtHMSO and prior PLM-guided sequence optimization methods, particularly in terms of search dynamics, constraint formulation, and theoretical justification of the heuristic strategy.

2. The experimental evaluation demonstrates improved functional performance; however, it is unclear whether the benchmarks sufficiently cover diverse protein families and functional regimes. Since protein sequence design methods may overfit to specific fitness landscapes or predictive models, further validation across heterogeneous datasets and experimentally validated fitness landscapes would strengthen the generalizability claims. Additionally, it would be valuable to assess robustness under varying fitness model qualities or noisy predictors.

3. ProtHMSO incorporates evolutionary priors to limit the search space, but this paper does not clarify whether structural stability or folding consistency is explicitly evaluated. Multi-site mutations can introduce epistatic effects that destabilize native conformations. Including structural validation metrics (e.g., predicted folding stability, structural deviation, or AlphaFold confidence scores) would provide stronger evidence that the generated variants maintain biophysical plausibility alongside improved fitness.

**********

**Have the authors made all data and (if applicable) computational code underlying the findings in their manuscript fully available?**

The PLOS Data policy requires authors to make all data and code underlying the findings described in their manuscript fully available without restriction, with rare exception (please refer to the Data Availability Statement in the manuscript PDF file). The data and code should be provided as part of the manuscript or its supporting information, or deposited to a public repository. For example, in addition to summary statistics, the data points behind means, medians and variance measures should be available. If there are restrictions on publicly sharing data or code —e.g. participant privacy or use of data from a third party—those must be specified.

Reviewer #1: None

Reviewer #3: None

Reviewer #4: Yes

**********

PLOS authors have the option to publish the peer review history of their article (what does this mean?). If published, this will include your full peer review and any attached files.

If you choose “no”, your identity will remain anonymous but your review may still be made public.

**Do you want your identity to be public for this peer review?**   For information about this choice, including consent withdrawal, please see our Privacy Policy.

Reviewer #1: No

Reviewer #3: **Yes:**   Guanghui Li

Reviewer #4: No

[NOTE: If reviewer comments were submitted as an attachment file, they will be attached to this email and accessible via the submission site. Please log into your account, locate the manuscript record, and check for the action link "View Attachments". If this link does not appear, there are no attachment files.]

**Figure resubmission:**

-->While revising your submission, we strongly recommend that you use PLOS’s NAAS tool (https://ngplosjournals.pagemajik.ai/artanalysis) to test your figure files. NAAS can convert your figure files to the TIFF file type and meet basic requirements (such as print size, resolution), or provide you with a report on issues that do not meet our requirements and that NAAS cannot fix.

After uploading your figures to PLOS’s NAAS tool - https://ngplosjournals.pagemajik.ai/artanalysis, NAAS will process the files provided and display the results in the "Uploaded Files" section of the page as the processing is complete. If the uploaded figures meet our requirements (or NAAS is able to fix the files to meet our requirements), the figure will be marked as "fixed" above. If NAAS is unable to fix the files, a red "failed" label will appear above. When NAAS has confirmed that the figure files meet our requirements, please download the file via the download option, and include these NAAS processed figure files when submitting your revised manuscript.

**Reproducibility:**

To enhance the reproducibility of your results, we recommend that authors of applicable studies deposit laboratory protocols in protocols.io, where a protocol can be assigned its own identifier (DOI) such that it can be cited independently in the future. Additionally, PLOS ONE offers an option to publish peer-reviewed clinical study protocols. Read more information on sharing protocols at https://plos.org/protocols?utm_medium=editorial-email&utm_source=authorletters&utm_campaign=protocols

# Author comment: Author response to Decision Letter 2

**DOI:** 10.1371/journal.pcbi.1014365.r004

8 Apr 2026

Attachment Submitted filename: Response Letter for ProtHMSO-updata.docx

# Peer review: Decision Letter 2

**DOI:** 10.1371/journal.pcbi.1014365.r005

**Authors:** Yanbu Guo

1 May 2026

PCOMPBIOL-D-25-01541R2

Heuristic Multi-site Optimization for Protein Sequence Design using Masked Protein Language Models

PLOS Computational Biology

Dear Dr. Chen,

Thank you for submitting your manuscript to PLOS Computational Biology. After careful consideration, we feel that it has merit but does not fully meet PLOS Computational Biology's publication criteria as it currently stands. Therefore, we invite you to submit a revised version of the manuscript that addresses the points raised during the review process.

Please submit your revised manuscript by Jul 01 2026 11:59PM. If you will need more time than this to complete your revisions, please reply to this message or contact the journal office at ploscompbiol@plos.org. When you're ready to submit your revision, log on to https://www.editorialmanager.com/pcompbiol/ and select the 'Submissions Needing Revision' folder to locate your manuscript file.

Please include the following items when submitting your revised manuscript:

* A letter that responds to each point raised by the editor and reviewer(s). You should upload this letter as a separate file labeled 'Response to Reviewers'. This file does not need to include responses to formatting updates and technical items listed in the 'Journal Requirements' section below.

* A marked-up copy of your manuscript that highlights changes made to the original version. You should upload this as a separate file labeled 'Revised Manuscript with Track Changes'.

* An unmarked version of your revised paper without tracked changes. You should upload this as a separate file labeled 'Manuscript'.

If you would like to make changes to your financial disclosure, competing interests statement, or data availability statement, please make these updates within the submission form at the time of resubmission. Guidelines for resubmitting your figure files are available below the reviewer comments at the end of this letter.

We look forward to receiving your revised manuscript.

Kind regards,

Yanbu Guo, Ph.D.

Academic Editor

PLOS Computational Biology

Shihua Zhang

Section Editor

PLOS Computational Biology

**Reviewers' comments:**

Reviewer's Responses to Questions

**Comments to the Authors:**

**Please note here if the review is uploaded as an attachment.**

Reviewer #1: The authors have addressed all the points raised.

Reviewer #3: The authors have addressed the previous comments. No more comments.

Reviewer #4: This paper proposes a heuristic searching framework called ProtHMSO to address the challenges of combinatorial space explosion and the structural instability caused by traditional random mutations in protein sequence design. ProtHMSO leverages the context-aware capabilities of a pre-trained protein language model to guide heuristic searches by predicting amino acid substitutions that conform to evolutionary laws and biophysical priors. Experiments on benchmarks demonstrate that this method significantly outperforms traditional random mutation strategies and accelerates classical optimization algorithms in improving protein functionality, activity, and maintaining structural stability.

This paper is novel and well presented. However, there are a few minor issues that should be addressed before publication.

1. The paper mentions that ProtHMSO can implicitly capture synergistic inter-residue epistasis zero-shot, but lacks a concrete explanation of the underlying principles. It is recommended to add a brief theoretical explanation to the Methodology or Discussion section.

2. ProtHMSO is further applied to replace the exploration strategies in genetic algorithms (GAs) and Monte Carlo tree search (MCTS) for improving their convergence efficiency. The default optimization strategies are modified. GA-HMSO adopts a dynamic crossover and mutation rate, while MCTS-HMSO employs group-based UCB values. It is suggested to clarify the underlying philosophy behind these modifications.

3. The literature review should be updated to cover the latest works in the field, such as the diffusion-based protein sequence generation methods (EvoDiff) and reinforcement learning-based methods for fine-tuning protein language models.

4. This paper also needs to clarify the practical application scenarios and transformation potential, such as how to accelerate the protein design process.

**********

**Have the authors made all data and (if applicable) computational code underlying the findings in their manuscript fully available?**

The PLOS Data policy requires authors to make all data and code underlying the findings described in their manuscript fully available without restriction, with rare exception (please refer to the Data Availability Statement in the manuscript PDF file). The data and code should be provided as part of the manuscript or its supporting information, or deposited to a public repository. For example, in addition to summary statistics, the data points behind means, medians and variance measures should be available. If there are restrictions on publicly sharing data or code —e.g. participant privacy or use of data from a third party—those must be specified.

Reviewer #1: None

Reviewer #3: Yes

Reviewer #4: None

**********

PLOS authors have the option to publish the peer review history of their article (what does this mean?). If published, this will include your full peer review and any attached files.

If you choose “no”, your identity will remain anonymous but your review may still be made public.

**Do you want your identity to be public for this peer review?**   For information about this choice, including consent withdrawal, please see our Privacy Policy.

Reviewer #1: No

Reviewer #3: **Yes:**   Guanghui Li

Reviewer #4: No

[NOTE: If reviewer comments were submitted as an attachment file, they will be attached to this email and accessible via the submission site. Please log into your account, locate the manuscript record, and check for the action link "View Attachments". If this link does not appear, there are no attachment files.]

**Figure resubmission:**

-->While revising your submission, we strongly recommend that you use PLOS’s NAAS tool (https://ngplosjournals.pagemajik.ai/artanalysis) to test your figure files. NAAS can convert your figure files to the TIFF file type and meet basic requirements (such as print size, resolution), or provide you with a report on issues that do not meet our requirements and that NAAS cannot fix.

After uploading your figures to PLOS’s NAAS tool - https://ngplosjournals.pagemajik.ai/artanalysis, NAAS will process the files provided and display the results in the "Uploaded Files" section of the page as the processing is complete. If the uploaded figures meet our requirements (or NAAS is able to fix the files to meet our requirements), the figure will be marked as "fixed" above. If NAAS is unable to fix the files, a red "failed" label will appear above. When NAAS has confirmed that the figure files meet our requirements, please download the file via the download option, and include these NAAS processed figure files when submitting your revised manuscript.

**Reproducibility:**

To enhance the reproducibility of your results, we recommend that authors of applicable studies deposit laboratory protocols in protocols.io, where a protocol can be assigned its own identifier (DOI) such that it can be cited independently in the future. Additionally, PLOS ONE offers an option to publish peer-reviewed clinical study protocols. Read more information on sharing protocols at https://plos.org/protocols?utm_medium=editorial-email&utm_source=authorletters&utm_campaign=protocols

# Author comment: Author response to Decision Letter 3

**DOI:** 10.1371/journal.pcbi.1014365.r006

10 May 2026

Attachment Submitted filename: Response Letter for ProtHMSO-R3.docx

# Peer review: Decision Letter 3

**DOI:** 10.1371/journal.pcbi.1014365.r007

**Authors:** Yanbu Guo

26 May 2026

Dear Prof. Chen,

We are pleased to inform you that your manuscript 'Heuristic Multi-site Optimization for Protein Sequence Design using Masked Protein Language Models' has been provisionally accepted for publication in PLOS Computational Biology.

Before your manuscript can be formally accepted you will need to complete some formatting changes, which you will receive in a follow up email. A member of our team will be in touch with a set of requests.

Please note that your manuscript will not be scheduled for publication until you have made the required changes, so a swift response is appreciated.

IMPORTANT: The editorial review process is now complete. PLOS will only permit corrections to spelling, formatting or significant scientific errors from this point onwards. Requests for major changes, or any which affect the scientific understanding of your work, will cause delays to the publication date of your manuscript.

Should you, your institution's press office or the journal office choose to press release your paper, you will automatically be opted out of early publication. We ask that you notify us now if you or your institution is planning to press release the article. All press must be co-ordinated with PLOS.

Thank you again for supporting Open Access publishing; we are looking forward to publishing your work in PLOS Computational Biology.

Best regards,

Yanbu Guo, Ph.D.

Academic Editor

PLOS Computational Biology

Shihua Zhang

Section Editor

PLOS Computational Biology

***********************************************************

Reviewer's Responses to Questions

**Comments to the Authors:**

**Please note here if the review is uploaded as an attachment.**

Reviewer #1: The authors have addressed all the issues.

Reviewer #3: No more comments.

Reviewer #4: The revised version of the manuscript has satisfactorily addressed all of my concerns. I therefore recommend it for acceptance.

**********

**Have the authors made all data and (if applicable) computational code underlying the findings in their manuscript fully available?**

The PLOS Data policy requires authors to make all data and code underlying the findings described in their manuscript fully available without restriction, with rare exception (please refer to the Data Availability Statement in the manuscript PDF file). The data and code should be provided as part of the manuscript or its supporting information, or deposited to a public repository. For example, in addition to summary statistics, the data points behind means, medians and variance measures should be available. If there are restrictions on publicly sharing data or code —e.g. participant privacy or use of data from a third party—those must be specified.

Reviewer #1: None

Reviewer #3: Yes

Reviewer #4: None

**********

PLOS authors have the option to publish the peer review history of their article (what does this mean?). If published, this will include your full peer review and any attached files.

If you choose “no”, your identity will remain anonymous but your review may still be made public.

**Do you want your identity to be public for this peer review?**   For information about this choice, including consent withdrawal, please see our Privacy Policy.

Reviewer #1: No

Reviewer #3: **Yes:**   Guanghui Li

Reviewer #4: No

# Editor report: Acceptance letter

**DOI:** 10.1371/journal.pcbi.1014365.r008

**Authors:** Yanbu Guo

PCOMPBIOL-D-25-01541R3

Heuristic Multi-site Optimization for Protein Sequence Design using Masked Protein Language Models

Dear Dr Chen,

I am pleased to inform you that your manuscript has been formally accepted for publication in PLOS Computational Biology. Your manuscript is now with our production department and you will be notified of the publication date in due course.

The corresponding author will soon be receiving a typeset proof for review, to ensure errors have not been introduced during production. Please review the PDF proof of your manuscript carefully, as this is the last chance to correct any errors. Please note that major changes, or those which affect the scientific understanding of the work, will likely cause delays to the publication date of your manuscript.

Soon after your final files are uploaded, unless you have opted out, the early version of your manuscript will be published online. The date of the early version will be your article's publication date. The final article will be published to the same URL, and all versions of the paper will be accessible to readers.

For Research, Software, and Methods articles, you will receive an invoice from PLOS for your publication fee after your manuscript has reached the completed accept phase. If you receive an email requesting payment before acceptance or for any other service, this may be a phishing scheme. Learn how to identify phishing emails and protect your accounts at https://explore.plos.org/phishing.

Thank you again for supporting PLOS Computational Biology and open-access publishing. We are looking forward to publishing your work!

With kind regards,

Lilla Horvath

PLOS Computational Biology | Carlyle House, Carlyle Road, Cambridge CB4 3DN | United Kingdom ploscompbiol@plos.org | Phone +44 (0) 1223-442824 | ploscompbiol.org | @PLOSCompBiol
